# Magnetically Boosted Water‐Splitting Performance in Metallic Glasses

**DOI:** 10.1002/advs.75877

**Published:** 2026-05-27

**Authors:** Chaoqun Pei, Zheng‐Jie Chen, Yuyang Qian, Zhichao Lu, Dong Ma, Jiang Ma, Tao Feng, Jing Peng, Baoan Sun, Weihua Wang

**Affiliations:** ^1^ Institute of Physics Chinese Academy of Sciences Beijing China; ^2^ Shenzhen Key Laboratory of High‐Performance Nontraditional Manufacturing College of Mechatronics and Control Engineering Shenzhen University Shenzhen China; ^3^ Songshan Lake Materials Laboratory Dongguan Guangdong China; ^4^ Faculty of Materials Science and Energy Engineering Shenzhen University of Advanced Technology Shenzhen China; ^5^ Herbert Gleiter Institute of Nanoscience School of Material Science and Engineering Nanjing University of Science and Technology Nanjing China

**Keywords:** high current density, magnetic‐field enhancement, metallic glasses, periodic magnetic domain, water‐splitting

## Abstract

Developing high‐performance non‐precious metal electrocatalysts for hydrogen production is vital to combat the energy crisis. Metallic glasses (MGs), a promising class of novel materials, have emerged as a focal point in the search for efficient catalysts. However, the lack of long‐range order in the amorphous structure of MGs poses significant challenges for precisely tuning their catalytic performance. Herein, we report a strategy to boost water electrolysis performance efficiency by using an ultralow magnetic field in soft‐magnetic MG wires. Remarkably, a magnetic field of merely 100 Oe, two orders of magnitude lower than that required for crystalline catalysts (10000 Oe for CoFe_2_O_4_), can significantly enhance the OER activity of Fe‐, Ni‐, and Co‐based MGs. Among them, Ni_40_Fe_40_P_20_ metallic glass can achieve an unprecedented overall water‐splitting performance with a cell voltage of 1.51 V @ 1000 mA cm^−2^ by magnetic modulation, marking a significant breakthrough among all catalysts reported ever. Furthermore, we revealed that the mechanism of magnetic enhancement is associated with the spin polarization within the unique periodic magnetic domain structure on the circumferential surface of MG wires, which increases orbital hybridization and net spin density. This work provides a new route for designing and modulating the electrocatalytic properties in disordered materials.

## Graphical abstract

A strategy for boosting water electrolysis efficiency using ultralow magnetic fields on soft‐magnetic MG wires is reported. Ni40Fe40P20 metallic glass exhibits superior overall water‐splitting performance (1.51 V @ 1000 mA cm^−^^2^) at 100 Oe, enabled by spin polarization in periodic surface magnetic domains to enhance orbital hybridization and net spin density.

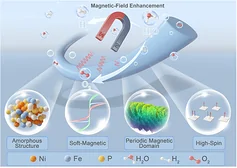

## 1 Introduction

Hydrogen energy is a cornerstone of the global transition to carbon‐neutral energy systems. Endowed with high energy density, abundance, and unparalleled environmental sustainability, it acts as a transformative solution to curtail reliance on carbon‐intensive fuels [1]. Among hydrogen production technologies, water electrolysis stands out as pivotal, owing to its ability to generate high‐purity hydrogen and seamless integration with renewable energy sources (e.g., solar, wind) [2, 3, 4, 5, 6]. However, the efficiency of this process is bottlenecked by the catalytic electrode, particularly the sluggish kinetics of the oxygen evolution reaction (OER) at the anode, which directly limits overall efficiency [7, 8]. Noble metal‐based catalysts (e.g., Ir and Ru) have long been the benchmark for OER due to their high catalytic activity and stability. Yet, their exorbitant costs and limited availability are significant drawbacks [9, 10, 11]. To address this, extensive efforts have focused on non‐precious metal catalysts (NPMCs) (notably Fe‐, Co‐, and Ni‐based materials), owing to their low cost and relatively high catalytic activity [12, 13, 14, 15, 16]. Despite these advantages, NPMCs still fail to meet the activity and stability requirements at high current densities for industrial‐grade electrolysis.

Metallic glasses (MGs), a class of materials defined by their disordered atomic structure, have emerged as promising next‐generation catalysts. Unlike conventional crystalline materials, their amorphous structure confers two unique catalytic advantages: a high density of unsaturated coordination sites (active centers) and reduced activation barriers for reaction intermediates, both of which boost catalytic performance [17, 18, 19, 20]. Furthermore, the short‐range disorder in MGs breaks lattice symmetry, enriching spin‐state distribution and enhancing spin‐charge coupling. These features are particularly beneficial for spin‐dependent catalytic reactions (such as OER), where electron spin polarization is critical [21]. For example, Jia et al. reported a defect‐rich high‐entropy MG (FeCoNiB_0.75_)_97_Pt_3_) that achieves ultralow overpotential and long‐term durability (>200 h at 100 mA cm^−2^) with just 3 at.% of Pt [22]. Similarly, a Ni‐P nanostructured MG with a heterogeneous structure exhibited exceptional urea oxidation performance (1.36 V at 10 mA cm^−2^, Tafel slope of 13 mV dec^−1^) among Ni‐based alloys [23]. Despite these advances, a critical bottleneck persists: the inherent atomic disorder of MGs makes precise regulation of their catalytic performance extremely challenging. Existing strategies like thermal cycling [24], cooling‐rate adjustment [25, 26], and severe plastic deformation [27] aim to modulate the energy state of MGs to optimize activity. However, these methods suffer inherent flaws: negligible efficacy in tuning active sites, excessively high energy consumption, and irreversible catalyst structural damage. Compositional regulation, a common strategy to tailor active sites, is highly sensitive to MG glass‐forming ability (GFA). Minor compositional adjustments alone can compromise amorphous structure integrity and impair catalytic performance. Therefore, how to develop a facile and efficient strategy to optimize the MGs catalytic activity, particularly at high current densities, remains an urgent, unresolved challenge.

Here, we address this critical gap by reporting that ultralow magnetic field modulation of water electrolysis performance in soft‐magnetic metallic glass wires (MGWs) with periodic domain structures. We found that applying a tiny magnetic field (100 Oe), two orders of magnitude lower than that required for traditional catalysts (e.g., 10000 Oe for CoFe_2_O_4_), significantly boosts the OER performance in Fe‐, Ni‐, and Co‐based soft‐magnetic MGWs. Notably, Ni_40_Fe_40_P_20_ (NiFeP) MGWs exhibit an exceptional magnetic‐enhanced OER performance with a prominent overpotential attenuation, and record‐low overall water‐splitting cell voltages of 1.51 V (@ 1000 mA/cm^2^), which surpass all catalysts at high‐current density reported so far. Mechanistically, we elucidate that the magnetic‐enhanced catalytic performance arises from spin polarization, which improves orbital hybridization and increases net spin density. Crucially, the periodic domain structure of NiFeP MGWs enables precise modulation of this enhancement, providing a controllable route to optimize MG catalytic performance. Our work harnesses periodic magnetic domain structure in soft‐magnetic MG to unlock high‐efficiency catalysis at industrial‐relevant current densities under ultralow magnetic fields.

## 2 Results

**FIGURE 1:**
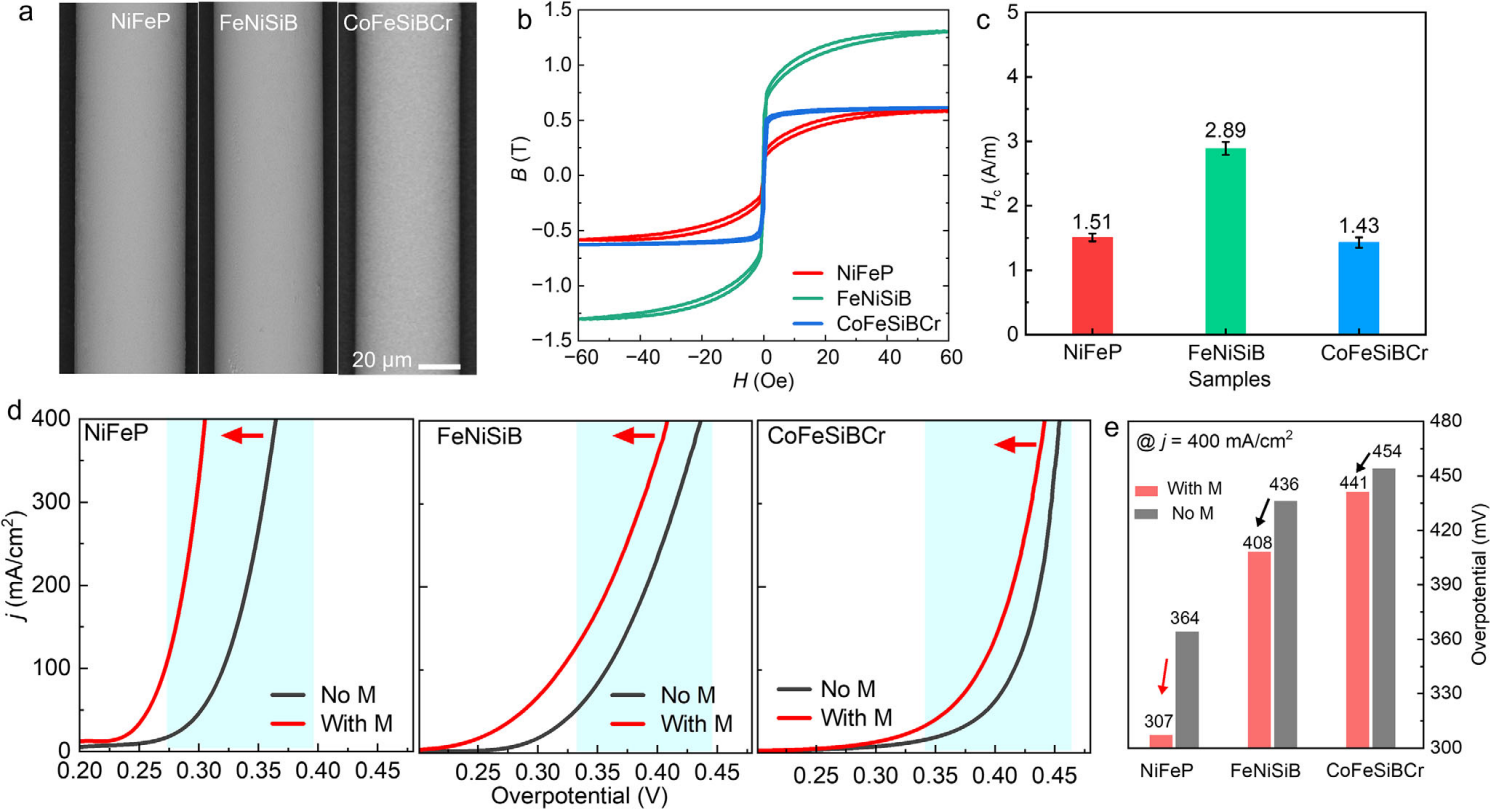
Soft‐magnetic properties and magnetic‐enhanced OER performance in different MGWs. (a) SEM characterization of NiFeP, FeNiSiB, and CoFeSiBCr MGWs. (b) Hysteresis loops of NiFeP, FeNiSiB, and CoFeSiBCr MGWs. (c) Coercivity (Hc) of NiFeP, FeNiSiB, and CoFeSiBCr MGWs. (d) LSV curves of various MGWs under a magnetic field (M = 100 Oe). (e) Statistics of overpotentials at the current density (j) of 400 mA/cm2 under a magnetic field (M = 100 Oe).

To investigate the electro‐catalytic water‐splitting performance under the influence of magnetic fields, we systematically studied the OER performance of magnetic MGWs. A schematic illustration of the electro‐catalytic testing of MGWs under the influence of a magnetic field is shown in Figure S1. The intensity of the magnetic field, spanning ± 100 Oe, emanated by the Helmholtz coil, is modulated by the applied direct current. The detailed magnetic field device can be referred to in Figure S2. The OER performance of MGWs was conducted using a three‐electrode electrolytic cell in the alkaline solution with 1 m KOH (Figure S2b, c). Three types of magnetic MGWs (NiFe‐based (NiFeP), Fe‐based (FeNiSiB), and Co‐based (CoFeSiBCr)) were fabricated by utilizing an advanced glass‐coated technique [28], which can continuously produce several kilometers of MGWs with a uniform diameter of ∼ 50 µm, as illustrated in Figure 1a and Figures S3, S4. Preceding the testing, all the MGWs underwent preparatory treatment in hydrofluoric acid to eliminate the surface glass layer. Figure 1a presents the surface morphology of the etched three magnetic MGWs as characterized by SEM, revealing a smooth and rounded surface devoid of the glass layer. The magnetic properties of the MGWs were characterized by hysteresis loop measurements (Figure 1b), revealing distinct low‐field magnetization behavior with saturation flux densities (*B*_s_) of ∼ 0.60 T (NiFeP), 1.30 T (FeNiSiB), and 0.61 T (CoFeSiBCr). Moreover, Figure 1c displays the obvious ultralow coercivity (*H*_c_) of approximately 1.51 A/m for NiFeP, 2.89 A/m for FeNiSiB, and 1.43 A/m for CoFeSiBCr (corresponding hysteresis loops see Figure S2b–d), respectively, highlighting their soft magnetic characteristics.

The OER performance of various types of MGWs in a magnetic field of 100 Oe (with M) is further investigated. Linear sweep voltammetry (LSV) curves of MGW with (red line) and without (black line) the application of a magnetic field (Figure 1d), reveal a notable enhancement in OER activity with M, particularly at high current densities (*j* > 100 mA/cm^2^). The NiFeP catalyst shows a substantial increase in catalytic activity, illustrated by a decrease in overpotential at 400 mA/cm^2^ by 57 mV compared to the nonmagnetic environment. Similar improvements were noted for FeNiSiB and CoFeSiBCr, with reduced overpotential (Δ*η*) of 28 and 13 mV, respectively (Figure 1e). We further investigated the OER performance of three MGWs under different magnetic field conditions, as shown in Figure S5. It was found that with the increase in magnetic field strength, the OER performance of all the tested MGWs exhibited an upward trend. We also provided the specific activity (mA cm^−2^_(__ECSA__)_) of all samples (including NiFeP, FeNiSiB, and CoFeSiBCr MGWs, in Figure S6) with and without a magnetic field, to eliminate the drawbacks of geometric area‐based current density, thus enabling a more accurate comparison of the intrinsic catalytic activity of different samples. In previous investigations, several crystalline magnetic catalysts (e.g., CoFe_2_O_4_ and NiZnFe_4_O_x_) have been reported to exhibit enhanced OER performance upon the application of an external magnetic field. For instance, CoFe_2_O_4_ and NiZnFe_4_O_x_ typically require magnetic fields of ∼10 000 and ∼4 500 Oe, respectively, to boost their catalytic activity [15, 16]. However, these crystalline magnetic NPMCs suffer from high coercivity, which necessitates the use of strong magnetic fields to modulate their catalytic performance. This not only leads to substantial energy consumption but also compromises overall efficiency, thereby limiting the broader, energy‐efficient application of such ferromagnetic NPMCs under magnetic field conditions. In contrast to these crystalline counterparts, the present work focuses on soft‐magnetic MGs materials with low coercivity. Notably, a mere 100 Oe magnetic field is sufficient to significantly enhance OER performance, which is only 1/100 the magnitude of that required for conventional crystalline materials. These results further demonstrate that the application of a magnetic field significantly enhances the OER performance of all tested MGW systems, suggesting that the integration of magnetic fields could be a validate approach to improve the efficiency of the electro‐catalytic water splitting process.

**FIGURE 2:**
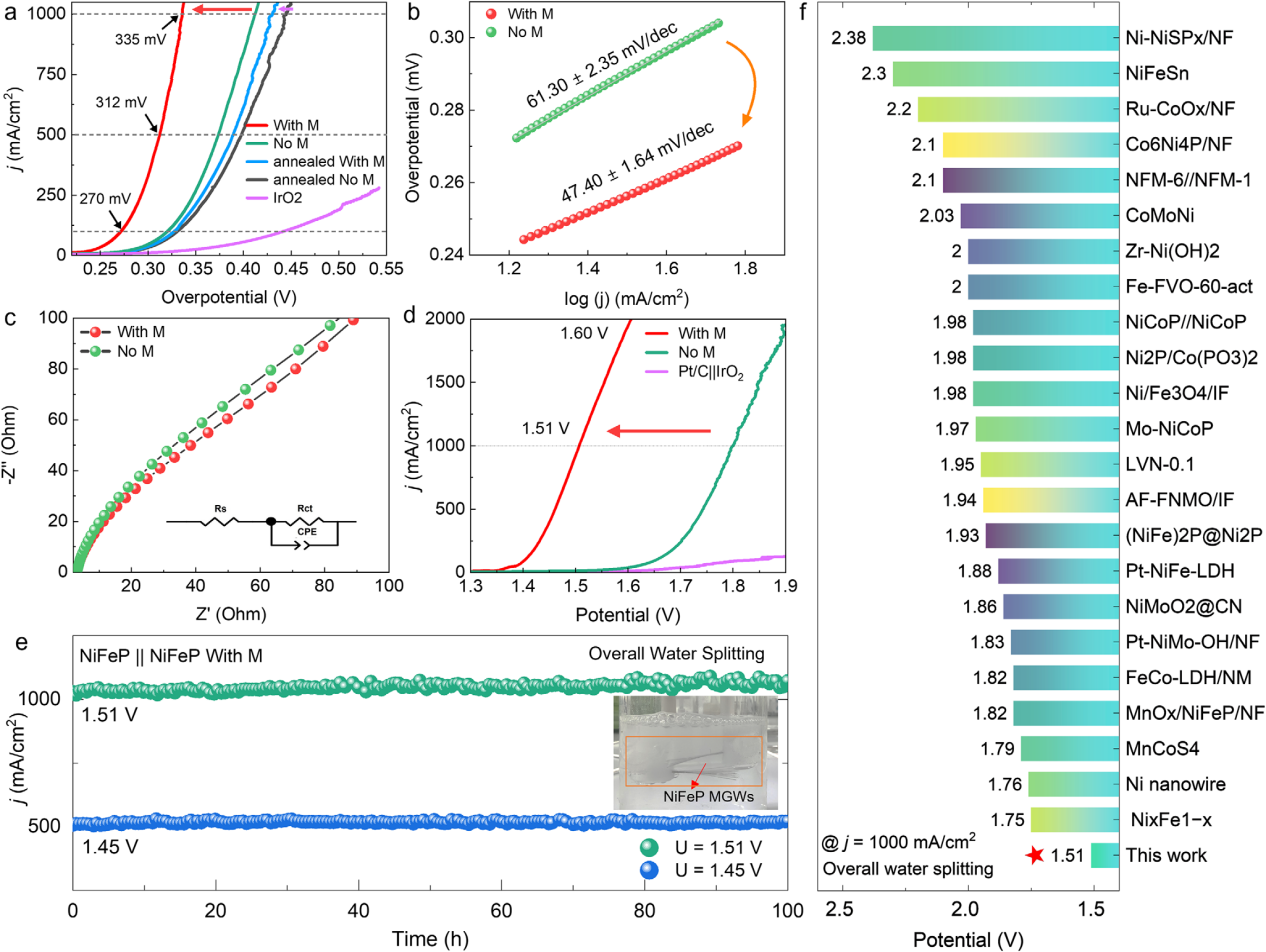
Water‐splitting performance of NiFeP MGWs under a magnetic field. a, LSV curves of NiFeP MGWs with high current density (j = 1000 mA/cm2) under a magnetic field on OER. b, Tafel slopes and c, EIS of NiFeP MGWs with and without a magnetic field. d, LSV curve of NiFeP || NiFeP device for overall water‐splitting in 1 m KOH under a magnetic field with a scanning rate of 5 mV s−1. e, Long‐term stability test of the prepared electrocatalysts under a magnetic field at a constant potential of 1.45 and 1.51 V, respectively, for 100 h. f, Comparison of the cell voltage to drive 1000 mA/cm2 of the NiFeP || NiFeP under a magnetic field and the previously reported electrocatalysts at room temperature.

The aforementioned results demonstrated that NiFeP MGWs have a significant magnetic field‐enhanced OER effect, positioning them as a promising candidate for industrial applications. To explore their industrial feasibility, we conducted a comprehensive evaluation of their catalytic properties at high current densities (*j* > 1000 mA/cm^2^). Notably, NiFeP MGWs exhibited the most prominent improvement in OER performance with M. Therefore, we further investigated the performance at high current densities (*j* > 1000 mA/cm^2^) under magnetic fields, as shown in Figure 2a. LSV measurements compared the performance of NiFeP MGWs with and without magnetic fields to that of a commercial catalyst of IrO_2_. Initially, it was observed that NiFeP MGWs outperformed IrO_2_ by achieving a lower overpotential of 319 mV at *j* = 100 mA/cm^2^, in the absence of magnetic fields, which is related to the high energy state of the MGWs [25, 29]. Intriguingly, we observed that the magnetic field‐enhanced effect of NiFeP MGWs (red line) exhibited marginal improvements at low current densities but became significantly pronounced at high current densities. Specifically, the OER overpotential of NiFeP MGWs decrease from 319 mV (@ 100 mA/cm^2^), 373 mV (@ 500 mA/cm^2^) and 412 mV (@ 1000 mA/cm^2^) to 270 mV (@ 100 mA/cm^2^), 312 mV (@ 500 mA/cm^2^), 335 mV (@ 1000 mA/cm^2^), respectively, under magnetic fields (Figure 2a). We have conducted LSV measurements at a scan rate of 2 mV/s to ensure steady‐state‐like conditions, as shown in Figure S7. The results demonstrate that the LSV curves acquired at this scan rate yield more stable data at low current densities, whereas negligible differences are observed in the results at high current densities. Moreover, experimental observations further reveal that the magnetic field exerts a remarkable enhancement effect on catalytic performance, particularly at high current densities, while its enhancement effect is negligible at low current densities. To further probe the reaction kinetics, the Tafel slope of NiFeP MGWs analysis (Figure 2b) revealed a decrease in the slope from 61.30 ± 2.35 mV/dec (no M) to 47.40 ± 1.64 mV/dec (with M), which indicates the more favorable reaction kinetics under magnetic fields (Tafel slopes of other MGWs can be seen in Figure S8). Moreover, the electrochemical impedance spectroscopy (EIS) results in Figure 2c show the smaller diameters of semicircles on Nyquist plots and lower values of charge transfer resistance (*R*_ct_ ∼ 172.6) of OER with M than that of No M (*R*_ct_ ∼ 205.9) (Table S1), indicating accelerated reaction kinetics of NiFeP MGWs during the OER process under a magnetic field.

To confirm that these enhancements were due to intrinsic material properties rather than structural rearrangements during the OER process, we conducted further surface structure analysis. Energy‐dispersive x‐ray spectroscopy (EDS) results revealed no significant elemental changes (oxide generation) on the surfaces of NiFeP MGWs before and after the OER under a magnetic field (Figure S9a). Raman spectra with XPS results (Figure S10) also show no noticeable oxidation of NiFeP MGWs surface before and after the OER under a magnetic field (Figure S9b), suggesting that magnetic field‐induced enhancements were independent of surface structural changes. We further examined the hydrogen evolution reaction (HER) performance under magnetic field conditions, and identified the identical trend to the OER process that the magnetic field affords a remarkable enhancement in its HER activity, as displayed in Figure S11. Emboldened by these results, we proceeded to assemble an alkaline electrolyzer using NiFeP MGWs electrocatalysts individually as the anode and cathode (Figure 2d). The electrolyzer exhibited an ultralow cell voltage of a mere 1.51 V at a current density of 1000 mA/cm^2^, and only 1.60 V at a current density of 2000 mA/cm^2^ (with 80% IR compensation, Figure S12), showcasing a marked superiority over the benchmark Pt/C || IrO_2_ configuration (purple line) (Figure S13). Moreover, a 100 h stability test under a magnetic field exhibited sustained stability with minimal current density fluctuations at two distinct constant potentials of 1.45 and 1.51 V, highlighting the catalyst's remarkable stability and durability in Figure 2e. In comparison with other non‐noble metal‐based electrocatalysts at a high current density of 1000 mA/cm^2^ (Figure 2f), the activity of NiFeP || NiFeP MGWs electrocatalysts under a magnetic field vastly surpasses that of the majority of non‐noble metal‐based electrocatalysts. Collectively, these findings underscore the exceptional overall water‐splitting performance and durability of our designed NiFeP MGWs electrocatalysts under a magnetic field, particularly in the context of high current density, thereby laying a strong foundation for their potential utilization in industrial‐scale hydrogen production in magnetic field‐assisted water‐splitting applications.

**FIGURE 3:**
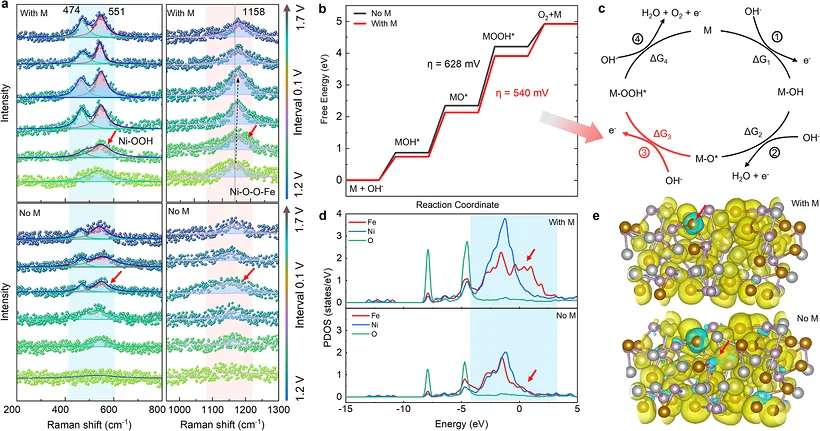
In situ Raman spectroscopy results and corresponding DFT calculations of NiFeP MGW under a magnetic field. a, In situ Raman spectra of the NiFeP MGW catalyst under a magnetic field obtained after holding at each potential (from 1.2 to 1.7 V, with the interval of 0.1 V) for 2 min. b, The free energy diagram of OER of NiFeP MGs before and after field application by DFT calculation. c, OER process with a four‐step electron transfer sequence NiFeP MGs before and after field application. d,e, The calculated PDOS and spin charge density before and after field application of NiFeP MGs.

To elucidate the mechanism underlying the enhanced OER performance of NiFeP MGWs under magnetic fields, we conducted in situ Raman experiments with and without magnetic field conditions, as depicted in Figure 3a. The testing voltages ranged from 1.2 to 1.7 V with intervals of 0.1 V, each held for 2 min to ensure data stability. In the process of without a magnetic field (No M), the Raman bands of NiFeP MGW showed no discernible peaks at 1.2 V, maintaining a stable metallic state. Upon increasing the voltage to 1.3 V, a weak Raman band appeared near 550 cm^−1^, possibly corresponding to the generation of Ni^2+^ [30]. Correspondingly, a minor fluctuation of the Raman band at 1150 cm^−1^ can be reasonably assigned to the superoxide ion (Ni─O─O─Fe stretching vibrations) [31, 32]. Further voltage increases to 1.5 V, there are two Raman bands at 474 and 551 cm^−1^, which indicates the transfer process of Ni^2+^ to Ni^3+^, accompanied by a prominent Raman peak at 1156 cm^−1^ (Ni─O─O─Fe stretching vibrations), signifying the active electron transfer characteristic of the third step of the OER process [33, 34, 35]. As the voltage reached 1.7 V, these peak positions remained stable, indicating sustained OER stability. In contrast, upon application of a magnetic field, these Raman bands shifted significantly. Notably, the Ni^2+^ to Ni^3+^ transition previously observed only between 1.4 and 1.5 V without a magnetic field was shifted to 1.3 V (474 and 551 cm^−1^), as indicated by the red arrow in Figure 3a) under the influence of a magnetic field. This shift corresponds to an accelerated process of Ni^3+^ generation, expediting the electron transfer in the third step of OER. Additionally, a distinct Raman band at 1158 cm^−1^ further supports this accelerated reaction mechanism. Furthermore, as the voltage increased to 1.4 V, the Raman bands became more pronounced with slight shifts, demonstrating a stabilized magnetic field‐driven acceleration in OER kinetics, which persisted up to 1.7 V. These findings suggest that a magnetic field facilitates Ni^3+^ generation kinetics, thereby enhancing the electron transfer process crucial for the improved OER efficiency. We also find that the undetectable Raman signals of NiFeP MGWs in ex situ measurements (Figure S9b) before and after reaction are mainly attributed to its intrinsic amorphous metallic bonding structure: metallic phases with delocalized valence electrons exhibit minimal polarizability change during vibration, leading to low Raman activity [32, 36], which is further confirmed by EDS and XPS results (Figure S9, S10) showing no obvious surface oxidation or elemental composition change. However, distinct Ni─OOH (active species) peaks in in situ Raman spectra (Figure 3a) above 1.4 V originate from the dynamic redox cycle of metallic Ni during OER: metallic Ni is oxidized to Ni─OOH (active species) under applied potential, and this transformation is reversible—Ni─OOH disappears when the reaction ceases, consistent with the ex situ results [15, 37]. Notably, the magnetic field modulates OER kinetics without inducing surface reconstruction. We further employed density functional theory (DFT) calculations to elucidate the mechanism of enhanced OER performance in amorphous NiFeP catalyst under a magnetic field by assessing the free energy and electronic structure, as illustrated in Figure 3b–e. Using NiFeP MG as a model system, we investigated the effects of a magnetic field on catalytic performance. Remarkably, the overpotential at the NiFeP active site decreased by 88 mV (from 628 to 540 mV) in the presence of the magnetic field (in Figure 3b). This decrease suggests that the magnetic field makes the active sites thermodynamically more favorable for OER. Moreover, the OER process was dissected into a four‐step electron transfer sequence (Figure 3c). The rate‐determining step (RDS) of the OER, specifically the transition from *O to *OOH, remained unchanged before and after the application of the magnetic field, in alignment with the observed Tafel slope (Figure 2b). To elucidate the microscopic changes induced by the magnetic field, we calculated the projected density of states (PDOS) and spin charge density before and after field application, as shown in Figure 3d (remarked by a red arrow). PDOS analysis indicated enhanced hybridization between the 3d orbitals of NiFe and the 2p orbitals of oxygen following magnetic field application. Additionally, the density of states for Fe shifted closer to the Fermi level, suggesting an increased availability of electrons at the Fermi level. This facilitates the greater electron participation in the reaction. The spin charge density analysis further revealed an increase in net spin after applying the magnetic field (yellow region in Figure 3e), promoting ferromagnetic exchange interactions. This increase in spin density likely lowers the energy barrier associated with the spin state transition from the singlet state H_2_O to the triplet state O_2_, thereby enhancing the overall catalytic activity of the NiFeP MGW catalyst.

**FIGURE 4:**
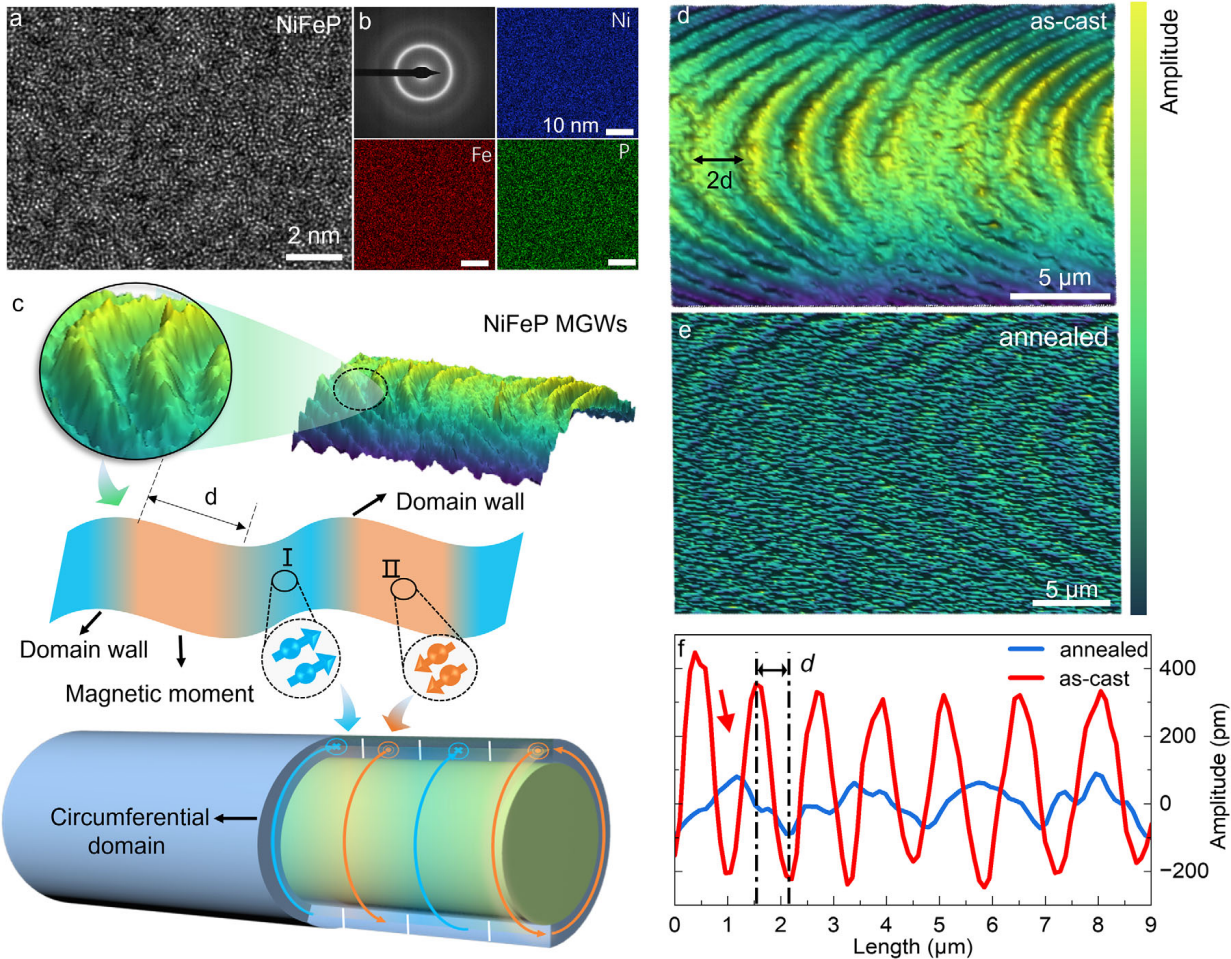
The micro and magnetic structure and of NiFeP MGW. a,b, The atomic structure characterization of NiFeP MGWs by HR‐TEM. c, 3D magnetic domain model of NiFeP MGWs by MFM. d,,e, Magnetic domain of as‐cast and annealed NiFeP MGWs by MFM. f, The intensity and period (d) of as‐cast and annealed NiFeP MGWs calculated from the magnetic domain.

The above results indicate that NiFeP MGWs magnetic catalyst will produce spin polarization during the magnetization process and enhance its catalytic performance, which may be related to its magnetic domain structure. Therefore, to clarify the influence of the magnetic domain structure of NiFeP MGWs on field‐enhanced catalytic performance, we undertook a detailed characterization of the magnetic properties of NiFeP MGWs in Figure 4. Fine x‐ray diffraction (XRD) analysis (Figure S14) revealed two diffuse peaks at ∼20^°^ and ∼35^°^, indicating that the amorphous structure of NiFeP MGWs (others MGWs XRD and corresponding DSC results are shown in Figures S15, S16). Furthermore, detailed structural characterization of the MGWs using high‐resolution transmission electron microscope (HR‐TEM, Figure 4a, b) and selected area electron diffraction (SAED) confirmed a disordered atomic structure characteristic of a typical amorphous state, with prominent amorphous diffuse rings, which is consistent with the structure depicted in Figures S14–S16. Energy dispersive x‐ray detector (EDX) analysis confirmed the uniform chemical composition distribution of the NiFeP with other MGWs prepared via glass‐coated technique, such as Figure 4b and Figure S17. We also supplemented post‐stability characterizations of NiFeP MGWs in Figure S18. Specifically, HR‐TEM and XRD results (Figure S18c,d,f) confirm that the post‐stability NiFeP MGWs maintain their disordered amorphous characteristics without a detectable crystalline phase. EDX (Figure S18e) further demonstrates uniform distribution of all constituent elements, with no obvious elemental segregation observed after the stability test. Regarding the surface composition evolution, XPS characterization (Figure S18g) shows a noticeable valence state shift of Ni species: the proportion of Ni^2^^+^ increases while that of Ni^0^ decreases after the stability test. This phenomenon may be attributed to the slight surface oxidation of the catalyst during long‐term immersion in the alkaline electrolyte for electrochemical measurements. Importantly, such minor oxidation does not impair the electrocatalytic performance of the samples under magnetic field conditions, which is supported by the stable current density during the stability test.

The magnetic domain structure of the NiFeP MGW were characterized by the magnetic force microscopy (MFM), as displayed in Figure 4c–f. Surprising, the domain structure shows a ring‐like periodic stripe pattern on the surface. The giant magnetoimpedance (GMI) profiles of MGWs (Figure S19) also exhibit a double‐peak shape in variation with the external field, similar to Co‐based MGWs [38, 39, 40]. This finding is in good agreement with the above analysis from the hysteresis loops and can be explained using the magnetization rotation model. Furthermore, magnetic domain structure of other systems of MGWs (Fe‐based and Co‐based) were also measured and unveiled a similar periodic circular stripe structure (in Figure S20), suggesting a correlation between this distinctive ring‐like periodic structure of MGWs and their magnetic properties, consistent with prior reports [38, 39, 40]. We observed distinctive crest and trough morphologies in the 3D magnetic domains visualized by MFM (Figure 4c), where the spatial transition from crest to trough corresponds to a single period (*d*) of the magnetic domain, as shown in the diagram in the middle of Figure 4c. The positions of these crests and troughs align precisely with the domain walls. Fixed region I (blue) and region II (orange) are opposite magnetic moments, depicted in the central diagram of Figure 4c [28, 41]. The regions between the crests and troughs reveal bamboo‐shaped circular magnetic domains, each oriented in opposing directions, as illustrated in Figure 4c,d. We further quantified the intensity and periodicity of the magnetic domain structures from Figure 4d, as illustrated in Figure 4f. It is evident that the magnetic domain intensity of the NiFeP MGW (∼380 pm, red line) has the most periodicity of magnetic domains of *d* ∼ 0.5 µm. As illustrated by the electron state distribution results depicted in Figure 3d,e, NiFeP MG has spin polarization under the action of magnetic field, which enhances 2p‐3d orbit hybridization and net spin. This polarization contributes to ferromagnetic exchange interactions, accelerating the process of the RDS in OER. Significantly, spin polarization augments the population of spin‐up‐oriented electrons in NiFeP, yielding a fixed magnetic moment. Therefore, the fixed magnetic moments mainly come from the contribution of the domain wall, especially the special circumferential periodic domain structure of NiFeP MGWs. The increase in the number of these domain walls causes a sharp increase in the number of magnetic moments in a fixed direction, thereby notably amplifying catalytic efficacy under magnetic field conditions of the amorphous NiFeP catalyst. The post‐stability MFM images (Figure S18a, b) reveal that the samples still retain distinct ring‐like stripe magnetic domain structures with clear periodicity after long‐term stability tests. The domain morphology is highly analogous to that of the as‐cast state, except for a slight attenuation in domain signal intensity.

We further analyzed the relationship between the periodic magnetic domain and the OER performance of NiFeP MGWs under a magnetic field. First, we weakened the magnetic properties of NiFeP MGWs by vacuum annealing, as shown in Figure 4e. It is obvious that NiFeP MGW remains in an amorphous state after annealing (Figure S21). However, the magnetic domain structure of annealed NiFeP MGW shows a significant change. Specifically, as shown in Figure 4f, a significant variation in the periodicity of magnetic domains is observed, with the *d* increasing from ∼0.5 µm in the as‐cast state to ∼2.5 µm after annealing (blue line), indicating an increase in domain width and a decrease in the periodicity after annealing. The magnetic domain strength decreases significantly after annealing, which is consistent with the decrease of *B*_s_ (∼0.28 T) in the *B–H* curve (Figure S22). Meanwhile, we also found that the OER properties changed correspondingly after annealing. Notably, the enhancement effect of OER performance of annealed samples under magnetic fields substantially attenuated, with overpotential decreasing from 330 mV (@ 100 mA/cm^2^), 397 mV (@ 500 mA/cm^2^) and 445 mV (@ 1000 mA/cm^2^) to 326 mV (@ 100 mA/cm^2^), 389 mV (@ 500 mA/cm^2^), 430 mV (@ 1000 mA/cm^2^), respectively (Figure 2a). Conversely, the Tafel slope of annealed NiFeP‐MGWs showed no significant reduction (from 66.72 mV/dec (no M) to 64.42 mV/dec (with M)) upon the application of magnetic fields (Figure S23). Therefore, the periodic domain structure of NiFeP MGW should be closely related to the catalytic performance under a magnetic field, that is, the MGW with the stronger periodic structure can produce the more excellent OER performance under a magnetic field. Our findings indicate that amorphous soft magnetic materials with a unique periodic magnetic domain structure can provide a new basis for regulating catalytic properties in various catalysts.

## 3 Conclusion

In summary, we report a prominent magnetic‐enhanced effect on high current water‐splitting performance via ultralow magnetic field applied in soft‐magnetic MGWs featuring periodic domain structures. We find that a weak magnetic field (100 Oe) can significantly and universally enhance OER performance in various magnetic MGWs systems, including Fe‐based, Ni‐based, and Co‐based MGWs. In contrast to these crystalline counterparts, soft‐magnetic MGs require only 1/100 the magnitude of the magnetic field, which is nonetheless sufficient to significantly enhance OER performance. Notably, this enhanced OER performance was particularly pronounced of NiFeP MGWs at high current densities, achieving a prominent overpotential attenuation of 77 mV at *j* = 1000 mA/cm^2^. Furthermore, NiFeP MGWs exhibit groundbreaking performance in overall water electrolysis under magnetic field conditions, demonstrating exceptionally low cell voltages of 1.51 and 1.60 V at high current densities of 1000 and 2000 mA/cm^2^, respectively. This remarkable achievement represents a significant advancement in the development of efficient electrocatalysts for industrial‐scale current density applications. Through DFT calculations, we also demonstrate that NiFeP MGs exhibit spin polarization, characterized by enhanced hybridization and increased net spin density under the influence of the magnetic field. This enhancement fosters stronger ferromagnetic exchange interactions and reduces the energy barrier associated with the spin state transition during the conversion of singlet state H_2_O to triplet state O_2_, which, in turn, enhances the overall catalytic activity of the NiFeP MGWs catalysts. Moreover, we further reveal that the catalytic activity of MGWs can be prominently regulated by their underlying periodic magnetic domain structure and domain intensity. As the periodicity and magnetism of the MGWs intensify, so does the effectiveness of the enhanced catalytic performance within the magnetic field. This work harnesses magnetic fields to induce highly efficient catalytic properties in ferromagnetic amorphous alloys, thus opening new avenues for enhancing their applications in catalysis and improving energy conversion efficiency.

## Author Contributions

**Zhichao Lu**: methodology, investigation. **Chaoqun Pei**: investigation, methodology, funding acquisition, writing – original draft, writing – review and editing. **Yuyang Qian**: investigation. **Dong Ma**: methodology, investigation, writing – review and editing. **Zheng – Jie Chen**: investigation, methodology. **Baoan Sun**: conceptualization, supervision, funding acquisition, writing – original draft, writing – review and editing. **Jing Peng**: methodology, investigation, funding acquisition, writing – review and editing. **Tao Feng**: supervision, funding acquisition, writing – review and editing, conceptualization. **Weihua Wang**: supervision, resources. **Jiang Ma**: investigation.

## Conflicts of Interest

The authors declare no conflicts of interest.

## Supporting information




**Supporting File**: advs75877‐sup‐0001‐SuppMat.docx.

## Data Availability

The data that support the findings of this study are available from the corresponding author upon reasonable request.
