# Supplementary material for: Magnetically Boosted Water‐Splitting Performance in Metallic Glasses
Source: Adv Sci (Weinh). 2026 May 27:e75877. Online ahead of print. doi: 10.1002/advs.75877 (PMC13335907; doi:10.1002/advs.75877)
Supplement: Supplementary file 1 — Supporting file: advs75877‐sup‐0001‐SuppMat.docx [file ADVS-9999-e75877-s001.docx]

**Supplementary Information for**

**Magnetically Boosted Water-Splitting Performance in Metallic Glasses**

**Chaoqun Pei^†^, Zheng-Jie Chen^†^, Yuyang Qian, Zhichao Lu, Dong Ma, Jiang Ma, Tao Feng^*^, Jing Peng^*^, Baoan Sun^*^, Weihua Wang**

^†^ These authors contributed equally to this work

* Corresponding Author:

Jing Peng, email: [jing.peng@siat.ac.cn](mailto:jing.peng@siat.ac.cn)

Tao Feng, email: tao.feng@njust.edu.cn

Baoan Sun, email: [sunba@iphy.ac.cn](mailto:sunba@iphy.ac.cn)

**Table of Contents:**

**Methods**

**Supplementary Figures** (Fig. S1-S23)

**Supplementary Table (**Table S1**)**

**Methods**

**Preparation of various MGWs**

Precursor synthesis employed high-purity metallic particles: Fe (99.99%, Zhongnuo Advanced Materials (Beijing) Technology Co., Ltd.), Ni (99.99%, Zhongnuo Advanced Materials (Beijing) Technology Co., Ltd.), Co (99.999%, Zhongnuo Advanced Materials (Beijing) Technology Co., Ltd.), Si (99.99%, Zhongnuo Advanced Materials (Beijing) Technology Co., Ltd.), Cr (99.99%, Zhongnuo Advanced Materials (Beijing) Technology Co., Ltd.), B (99.9%, Zhongnuo Advanced Materials (Beijing) Technology Co., Ltd.), and red phosphorus (≥99.999% metal basis, Aladdin). These raw materials were blended in specific atomic ratios for target compositions: 40:40:20 (Ni_40_Fe_40_P_20_), 55:20:10:15 (Fe_55_Ni_20_Si_10_B_15_), and 68:4.5:14.5:10:3 (Co_68_Fe_4.5_Si_14.5_B_10_Cr_3_). Precursor alloys were melted in a vacuum arc furnace (base pressure: 2×10^-4^ Pa) and cast into 5 mm-diameter rods via copper mold suction casting under a high-purity argon atmosphere. The alloy rods were then sectioned into 7 mm-long segments using electrical discharge machining, followed by surface oxide layer removal with abrasive papers. Metallic glass (MG) microwire fabrication was conducted in a vacuum chamber via inductive melting (single-turn copper coil, 400 kHz ultrahigh-frequency heating source), named Taylor–Ulitovsky (TU) method. Precursor ingots were melted into a molten pool, with a high-boron borosilicate glass tube guiding the melt—its tapered tip design facilitated wire drawing. Notably, the alloy’s melting temperatures need over the glass tube’s supercooled liquid region (~ 1120 K), inducing substantial softening of the viscous glass matrix. Upon application of a traction force, the molten alloy was drawn through the glass tube, solidified continuously by the fast-cooling system to form the amorphous structure, and wound onto a roller for practical applications. Traction speeds of 35–75 m min⁻^1^ produced microwires with a diameter of ~50 μm, as illustrated in Fig. S3.

**Electrochemical Testing Device Under Magnetic Field**

The schematic diagram of the magnetic field application device was showed in Fig. S1-S2 Specifically, a pair of Helmholtz coils integrated with a current source is employed to generate a tunable magnetic field ranging from 0 to 100 Oe. The key parameters of the Helmholtz coils are as follows: the number of turns is 340, the total resistance of the coils is 2.3 Ω, the inner diameter is 230 mm, the outer diameter is 330 mm, and the wire diameter of the winding is 2.0 mm. The current source is capable of supplying a current varying from 0 to 10 A. Both theoretical calculation and experimental characterization demonstrate that a current of 1 A can produce a magnetic field of 10.0 ± 0.02 Oe, with the linearity of the coils being 1%. Consequently, the coils can provide a magnetic field with a range of 100 ± 0.15 Oe. It should be noted that the coils underwent magnetic field calibration prior to factory delivery to ensure the accuracy of the generated magnetic field. Furthermore, we have also characterized the error associated with the uniform magnetic field distribution range at the center of the Helmholtz coils. Within a cubic region of 63 mm × 63 mm × 63 mm centered at the coil core, the magnetic field error is less than 1%, which ensures the magnetic field uniformity of the testing environment for MGWs. Fig. S2b depicts the operational configuration where a three-electrode electrolytic cell is placed at the center of the Helmholtz coils. We have provided the actual photo of the MGWs catalyst electrode, as shown in Fig. S2c.

**Structural characterizations**

X-ray diffraction (XRD) was conducted using a BRUKER D8 ADVANCE/DISCOVER instrument equipped with Cu-*K_α_* and Mo-*K_α_* radiation sources, with a wavelength of *λ* = 1.5418 Å (Germany), to elucidate the amorphous structures of the microwires. The morphological characteristics of the samples were examined employing a scanning electron microscope (SEM, IT500). Transmission electron microscopy (TEM) was carried out using a FEI Tecnai G2 F30 S-Twin operating at 300 kV, and the elemental distribution within the wires was analyzed through Energy Dispersive X-Ray Spectroscopy (EDX). In-situ Raman spectroscopy was performed using a Horiba LabRAM HR Evolution system, utilizing a laser with a wavelength of 532 nm, a grating condition of 600 gr/mm, and a laser spot diameter of 1298 nm. During the in-situ Raman spectroscopy testing, the voltages were systematically varied from 1.2 V to 1.7 V in 0.1 V increments, with each voltage maintained for 2 minutes to ensure data stability.

**Magnetic force microscope (MFM) measurement**

MFM measurements were performed using an Atomic Force Microscope (AFM, Oxford Instruments Asylum Research Cypher S) in tapping mode, where the cantilever was driven slightly below its natural resonance frequency to enhance oscillation amplitude sensitivity. A constant lift height of 100 nm was maintained for all samples prior to measurement. MFM images were obtained by mapping the resonance variations of a cantilever equipped with a perpendicularly magnetized tip as it interacted with the stray magnetic field of the samples. The amplitude and phase images exhibited similar features, with amplitude providing superior contrast. Instead of capturing discrete values, the average magnetic field near the probe was measured, which has negligible impact on the MFM images when the lift height exceeds the probe size. In this study, the MG microwires were secured to a horizontal substrate beneath a silicon probe with a hard magnetic coating (coercivity of 300 Oe, remanent magnetization of 300 emu/cm³). The probe featured a height of 10–15 μm, a tip radius of < 50 nm, and a force constant of 2.8 N/m.

**Electrochemical measurements**

After comprehensive characterization, both the oxygen evolution reaction (OER) and overall water-splitting were assessed in an alkaline solution (1 M KOH) to evaluate catalytic performance under varying magnetic fields (M =0 and 100 Oe). OER electrochemical measurements was conducted in a standard three-electrode setup and the overall water-splitting measurements was conducted in a standard double-electrode setup, utilizing an electrochemical workstation for catalytic analysis. In the standard three-electrode setup, the synthesized NiFeP MGWs electrode served as the working electrode, while a commercial platinum electrode acted as the counter electrode, and a Hg/HgO electrode was employed as the reference. The measured potentials were converted to the reversible hydrogen electrode (RHE) scale using the equation *E_RHE_* = *E_Hg/HgO_* + 0.059 *pH* + 0.098 V-*iR*. To ensure O_2_/H_2_O equilibrium at 1.23 V vs RHE during OER measurements, high-purity oxygen was bubbled through the electrolyte. Cyclic voltammetry (CV) at a scan rate of 100 mV s⁻¹ was performed for 20 cycles prior to linear sweep voltammetry (LSV) at 5 and 2 mV s⁻¹ to characterize the OER electro-catalytic activity of each sample. Electrochemical impedance spectroscopy (EIS) was conducted at the open circuit potentials of each sample, spanning frequencies from 100 kHz to 0.01 Hz with an AC voltage amplitude of 5.0 mV. Stability was assessed through chronoamperometry, recording current density versus time over a duration of 100 hours at a fixed potential.

**Molecular Dynamics (MD) Construction of Amorphous Models**

Amorphous structural models were generated by a melt–quench ab initio molecular dynamics (AIMD) protocol as implemented in VASP. A stoichiometric bulk supercell (≈108 atoms) was constructed and pre-relaxed, after which AIMD was performed in the NVT ensemble using a Nosé–Hoover thermostat with a 1.0 fs time step. The system was first equilibrated at 3000 K for 6 ps to erase the initial structural memory and remove long-range order, then cooled stepwise to 300 K with 4 ps equilibration at each intermediate temperature. During the melt–quench and subsequent relaxation, the evolution of “energy without entropy”, maximum atomic force, and external pressure versus ionic steps was monitored by parsing OUTCAR/OSZICAR outputs using an in-house script, ensuring both thermal equilibration and mechanical stability of the obtained structure. The amorphous nature of the resulting models was validated by the absence of long-range periodicity; therefore, no Miller-index facets were assigned, and all surface slabs used for subsequent energetics were generated by cleaving representative snapshots from the amorphous bulk.

**DFT calculations**

All calculations are based on density functional theory (DFT) with the Vienna Ab-initio Simulation Package (VASP) [1-2]. The exchange-correlation interactions were treated using the generalized gradient approximation (GGA) [3] combined with the Perdew-Burke-Ernzerhof (PBE) functional [4-5]. To account for d-electron Coulomb interactions in transition metals, the GGA+U method was applied [6], with Ueff values of 3.5 eV for Fe, 2.5 eV for Ni. A plane-wave basis set with 450 eV cutoff energy was used. Spin polarization effects were included, and the Monkhorst-Pack method [7] generated a 3 × 3 × 1 *K*-point. And convergence thresholds for atomic forces and energy were set at -0.01 eV/Å and 4 × 10^-4^ eV, respectively. Electrocatalytic activity was assessed by calculating the Gibbs free energy (Δ*G*) for each reaction step [8], using the formula: Δ*G* = Δ*E* + Δ*E*_ZPE_ - *T*Δ*S* – eU, where Δ*E* represents the adsorption energy, Δ*E*_ZPE_ accounts for zero-point energy differences, *T*Δ*S* is the entropy term, and U is the potential relative to the standard hydrogen electrode (SHE). The electron free energy was derived from H_2_. To simulate the influence of an external magnetic field, which aligns the spins in the catalyst, the "LNONCOLLINEAR" and "SAXIS" parameters were employed in VASP to direct the spin alignment [9].

References

[1] J. Hafner, *J. Comput. Chem.* **2008**, *29* (13), 2044.

[2] E. Wimmer, M. Christensen, V. Eyert, W. Wolf, D. Reith, X. Rozanska, C. Freeman, P. Saxe, E. Wimmer, M. Christensen, *J. Korean Ceram. Soc.* **2016**, *53* (3), 263.

[3] J. P. Perdew, K. Burke, M. Ernzerhof, *Phys. Rev. Lett.* **1996**, *77* (18), 3865.

[4] Z. Chen, J. Cao, L. Yang, W. Yin, X. Wei, *J. Phys. D: Appl. Phys.* **2018**, *51* (26), 265106.

[5] M. Ernzerhof, G. E. Scuseria, *J. Chem. Phys.* **1999**, *110* (11), 5029.

[6] C. Ren, S. Lu, Y. Wu, Y. Ouyang, Y. Zhang, Q. Li, C. Ling, J. Wang, *J. Am. Chem. Soc.* **2022**, *144* (28), 12874.

[7] K. Choudhary, F. Tavazza, *Comput. Mater. Sci.* **2019**, *161*, 300.

[8] A. Kulkarni, S. Siahrostami, A. Patel, J. K. Nørskov, *Chem. Rev.* **2018**, *118* (5), 2302.

[9] Z. Gao, G. Mao, S. Chen, Y. Bai, P. Gao, C. Wu, I. D. Gates, W. Yang, X. Ding, J. Yao, *Phys. Chem. Chem. Phys.* **2022**, *24* (5), 3460.

**Supplementary Figures and Table**

Fig. S1 Schematic diagram of catalytic testing with MGWs under a magnetic field condition.

Fig. S2 Schematic diagram of the magnetic field application device for MGWs electrocatalytic performance testing.

Fig. S3 Fig. S3 Schematic diagram of the preparation of MGWs.

Fig. S4 Schematic representation of MGWs extending over several kilometers with hysteresis loops.

Fig. S5 OER performances under different magnetic field conditions for NiFeP, FeNiSiB and CoFeSiBCr MGWs.

Fig. S6 The specific activity (mA cm^-2^₍_ECSA_₎) of all samples (including NiFeP, FeNiSiB and CoFeSiBCr MGWs) measured under the conditions with and without a magnetic field.

Fig. S7 LSV curves of NiFeP MGWs at different scan rates.

Fig. S8 Tafel slopes of FeNiSiB and CoFeSiBCr MGWs with and without magnetic field.

Fig. S9 The surface analysis of NiFeP MGW before and after OER under the magnetic field.

Fig. S10 XPS results of Ni element NiFeP MGWs before and after OER under magnetic field.

Fig. S11 HER polarization curves with/without magnetic field of NiFeP MGWs.

Fig. S12 Both the IR-compensated (80% IR correction) and uncompensated raw data for the overall water-splitting performance of NiFeP MGWs.

Fig. S13 EIS result of NiFeP || NiFeP MGWs during overall water-splitting under magnetic field.

Fig. S14 Fine XRD analysis results of NiFeP MGWs.

Fig. S15 XRD results of NiFeP, FeNiSiB and CoFeSiBCr MGWs.

Fig. S16 DSC results of NiFeP, FeNiSiB and CoFeSiBCr MGWs.

Fig. S17 SEM and corresponding EDS mapping results of NiFeP, FeNiSiB and CoFeSiBCr MGWs.

Fig. S18 Structure characterizations of post-stability of NiFeP MGWs.

Fig. S19 GMI result of NiFeP MGWs.

Fig. S20 3D-magnetic domain structure results of FeNiSiB and CoFeSiBCr MGWs.

Fig. S21 Fine-XRD results of as-cast and annealed NiFeP MGWs.

Fig. S22 B-H results of as-cast and annealed NiFeP MGWs.

Fig. S23 Tafel slopes of as-cast and annealed NiFeP MGWs with and without magnetic field.

Table S1 Fitted results for EIS spectra of NiFeP MGWs with and without magnetic fields.


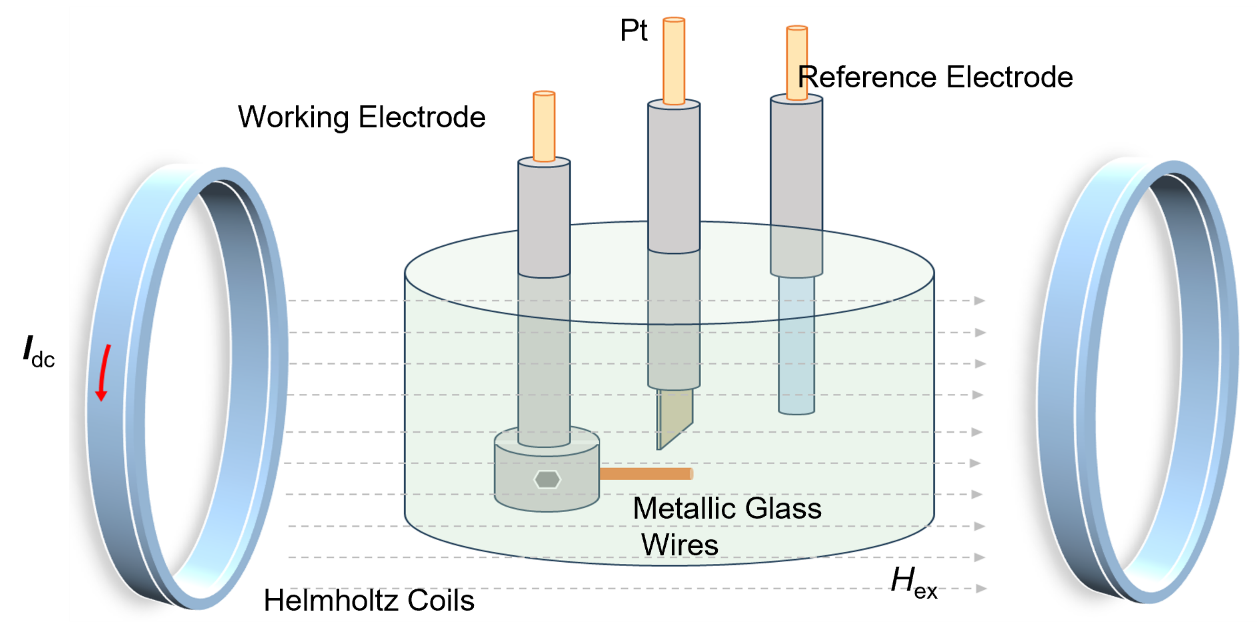


**Fig. S1 Schematic diagram of catalytic testing with MGWs under a magnetic field condition.**


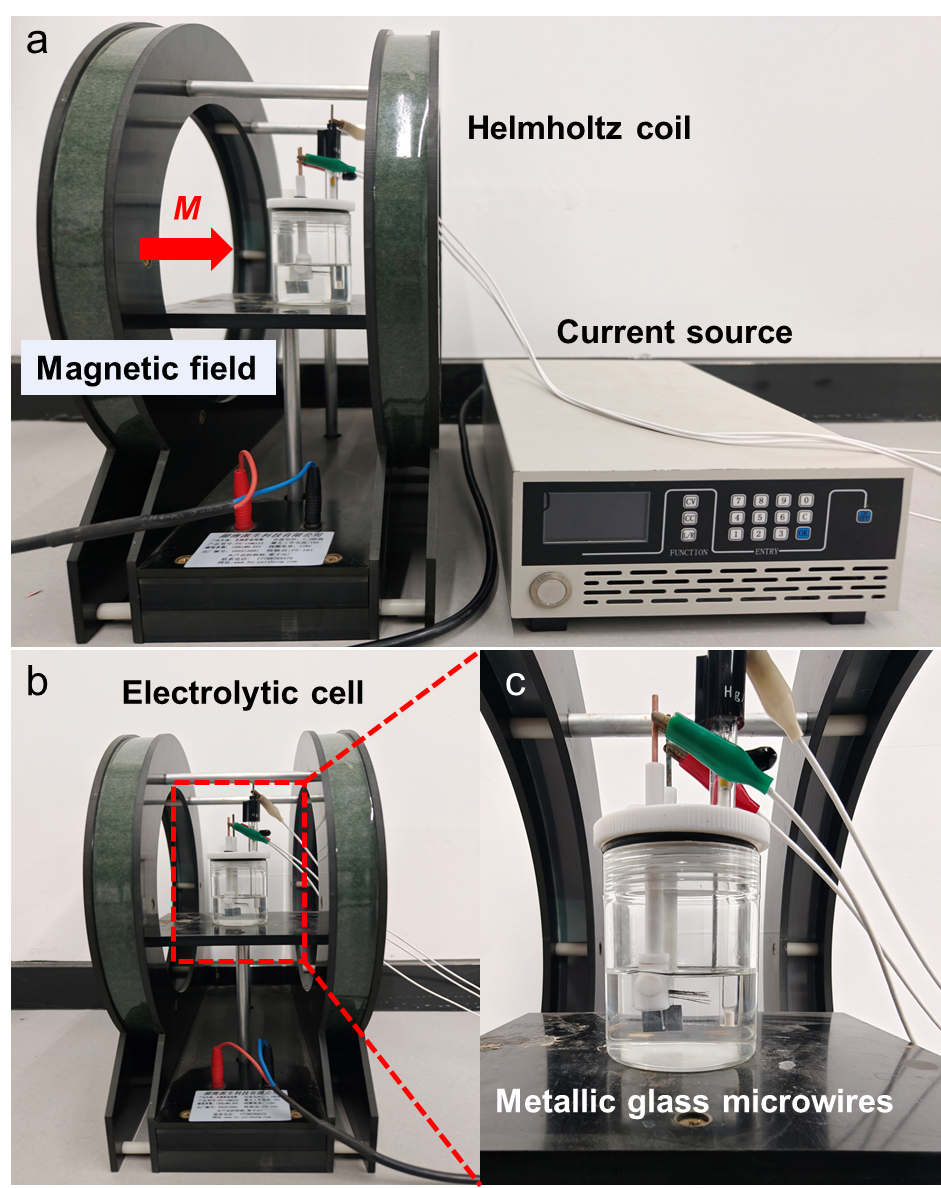


**Fig. S2 Schematic diagram of the magnetic field application device for MGWs electrocatalytic performance testing.**


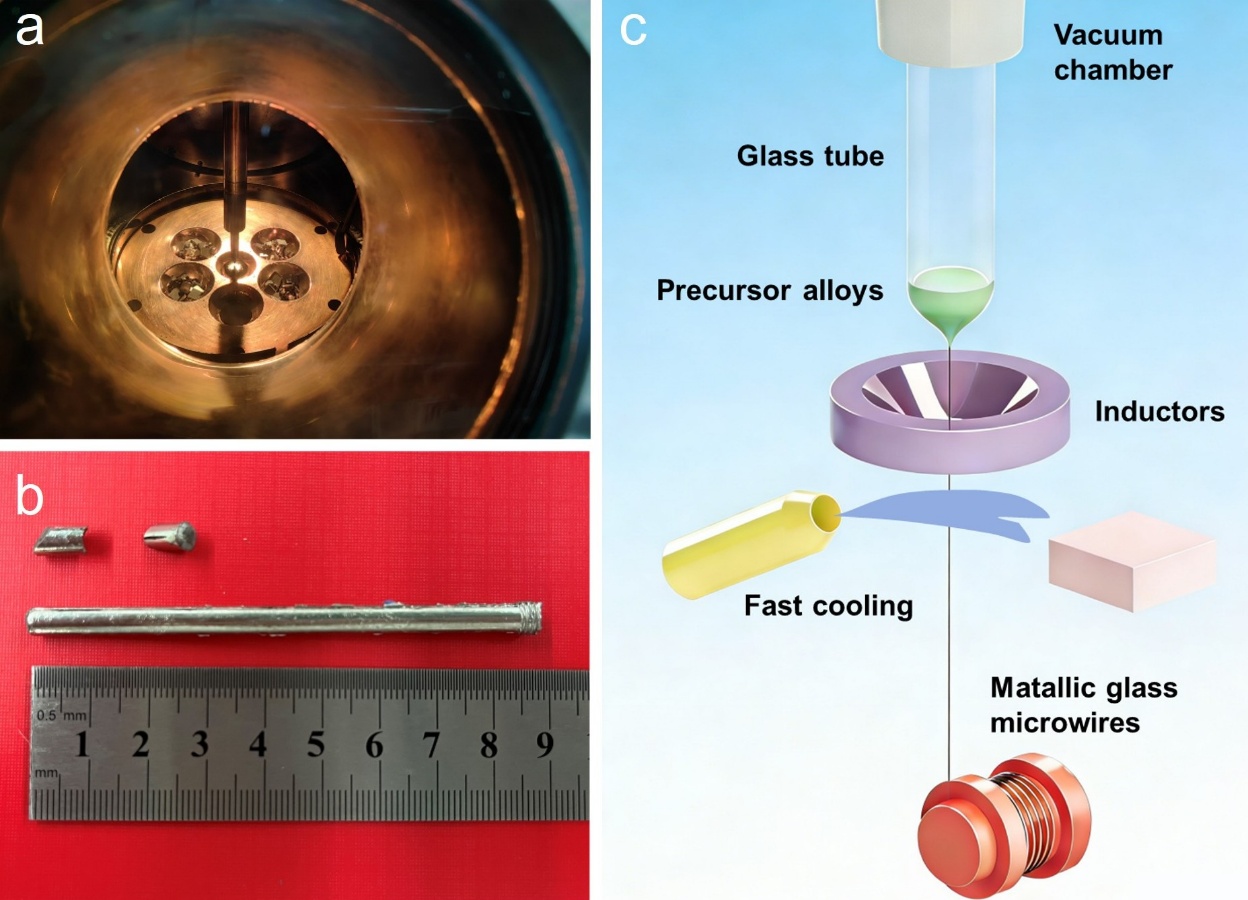


**Fig. S3 Schematic diagram of the preparation of MGWs.**


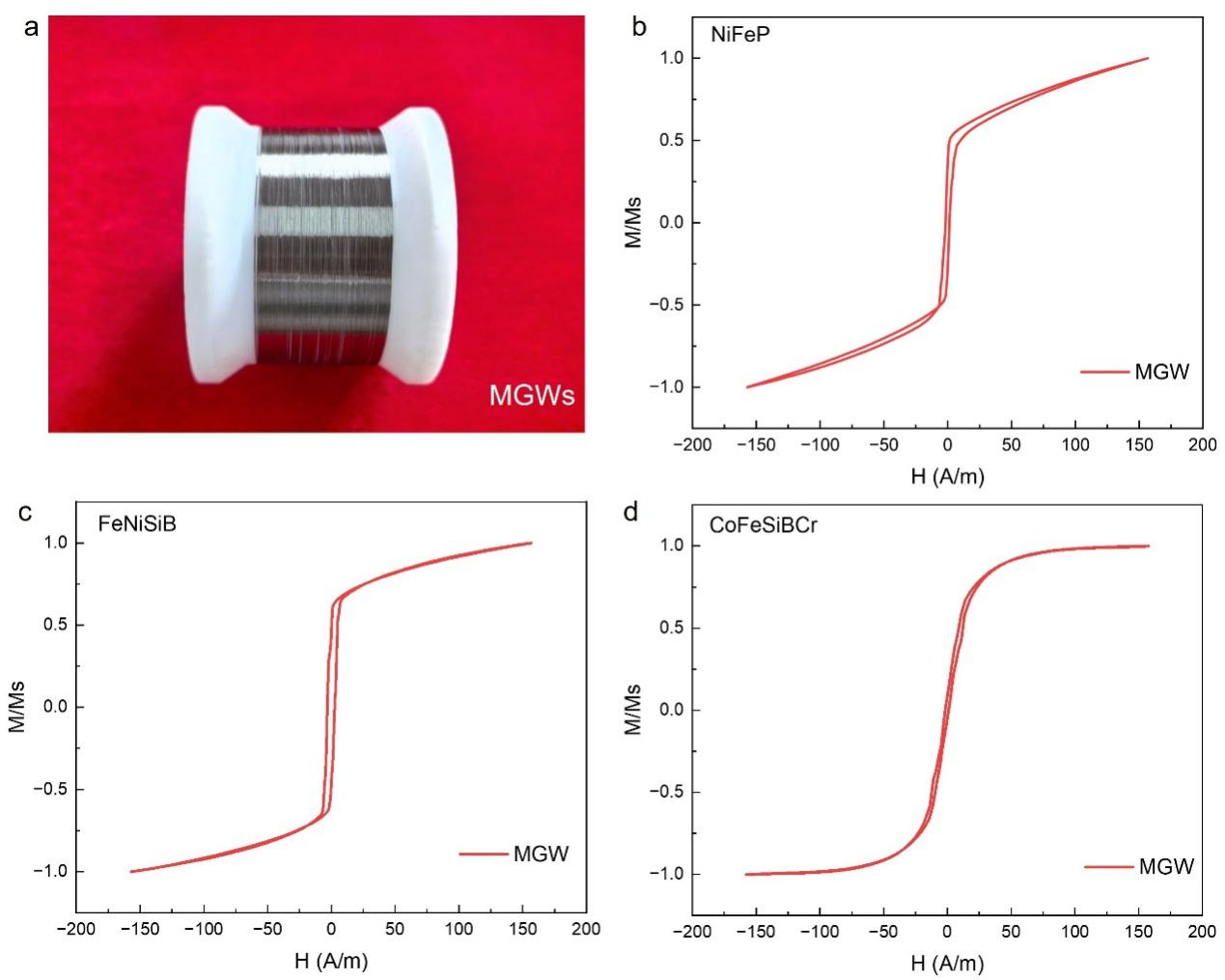


**Fig. S4 Schematic representation of MGWs extending over several kilometers with** **hysteresis loops.**


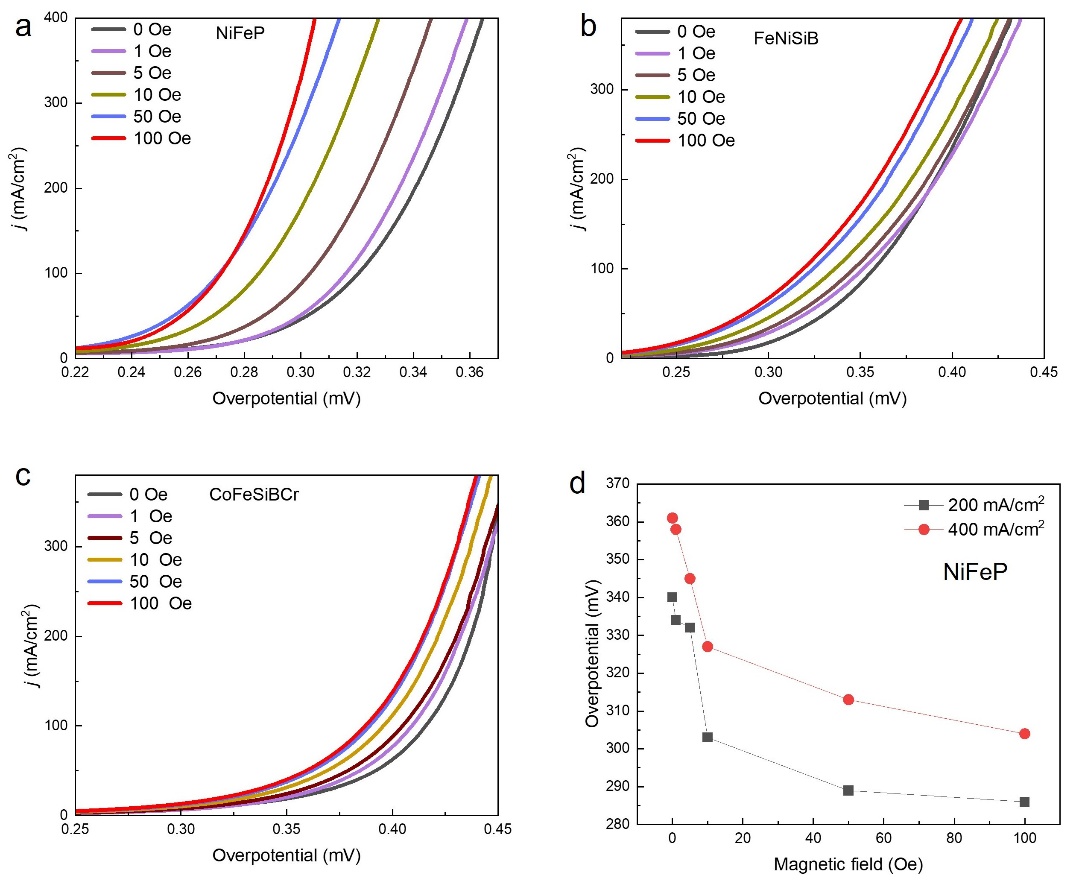


**Fig. S5 OER performances under different magnetic field conditions for NiFeP, FeNiSiB and CoFeSiBCr MGWs.**


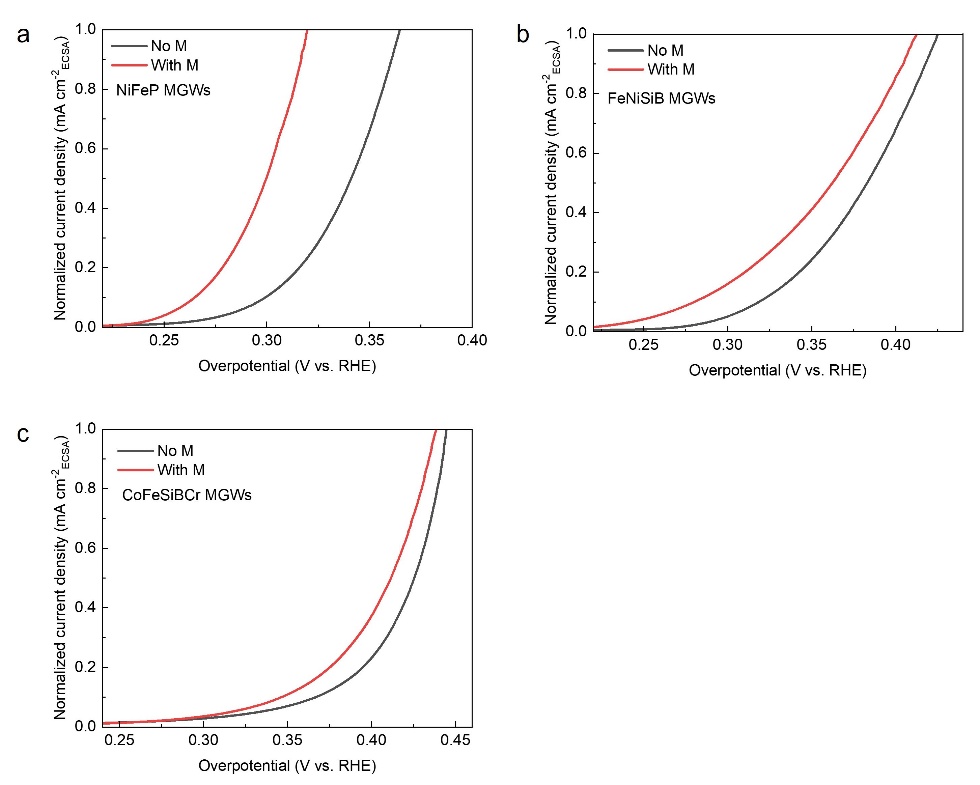


**Fig. S6 The specific activity (mA cm^-2^₍_ECSA_₎) of all samples (including NiFeP, FeNiSiB and CoFeSiBCr MGWs) measured under the conditions with and without a magnetic field.**


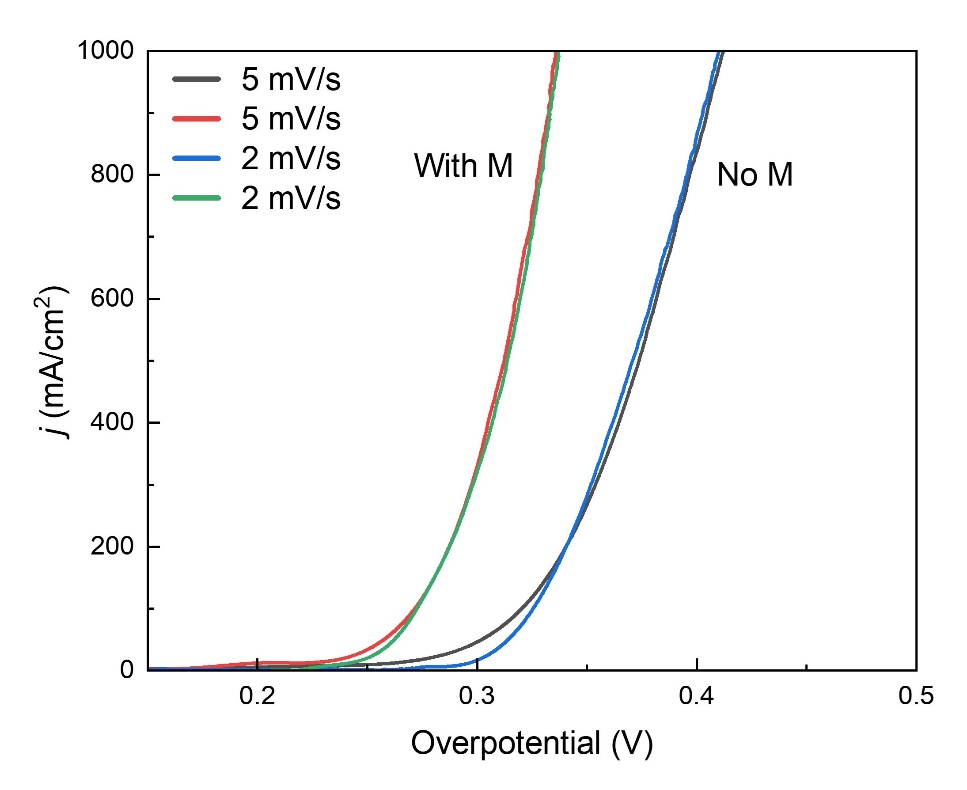


**Fig. S7 LSV curves of NiFeP MGWs at different scan rates.**


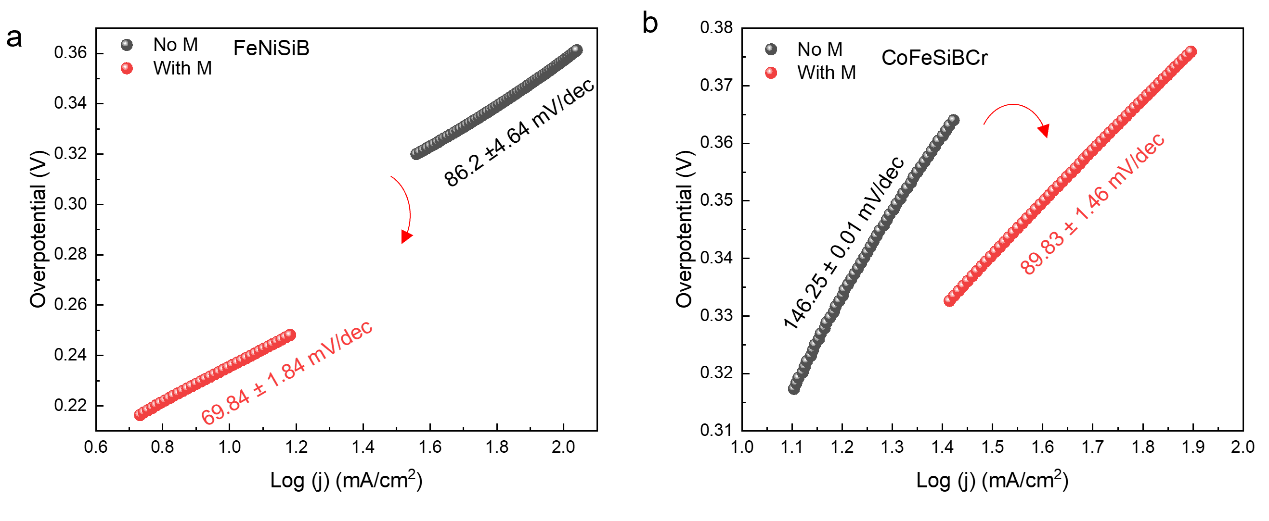


**Fig. S8 Tafel slopes of FeNiSiB and CoFeSiBCr MGWs with and without magnetic field. a, FeNiSiB MGWs. b, CoFeSiBCr MGWs.**


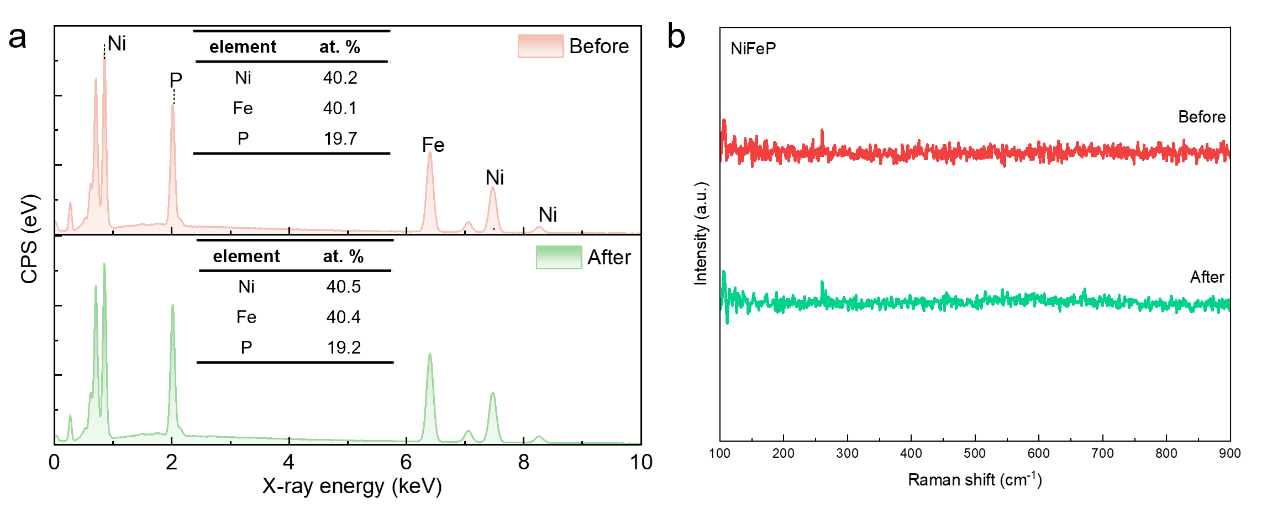


**Fig. S9 The surface analysis of NiFeP MGW before and after OER under the magnetic field. a**, EDS of surface structure of NiFeP MGWs before and after OER under magnetic field. **b**, Raman spectra of NiFeP MGWs before and after OER testing under a magnetic field.


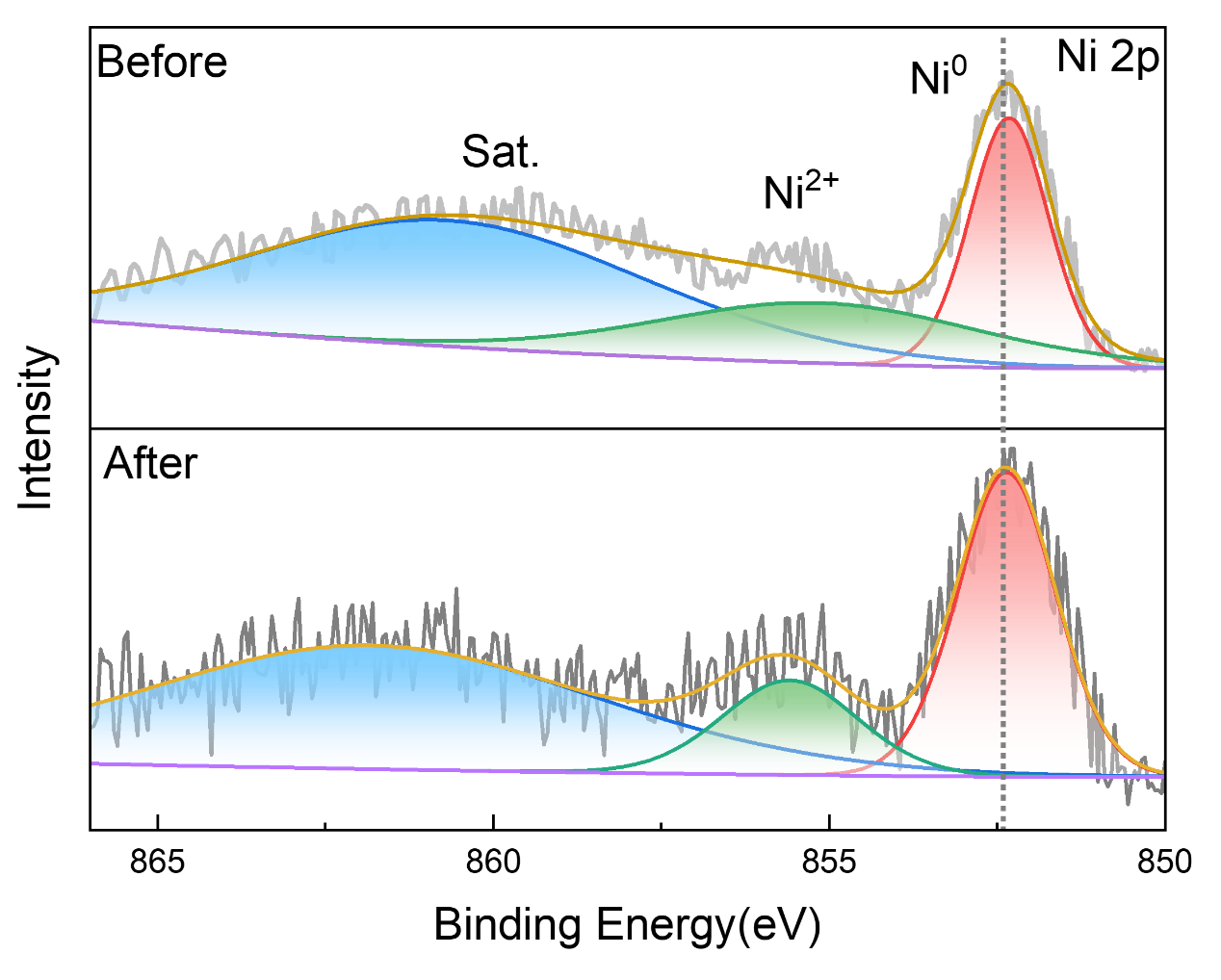


**Fig. S10 XPS results of Ni element NiFeP MGWs before and after OER under magnetic field.**


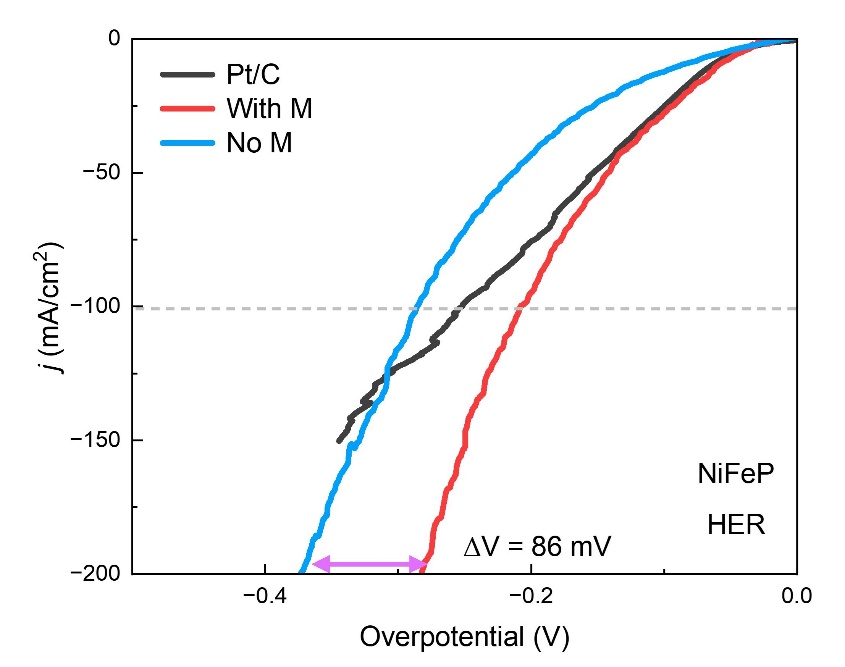


**Fig.S11 HER polarization curves with/without magnetic field of NiFeP MGWs.**


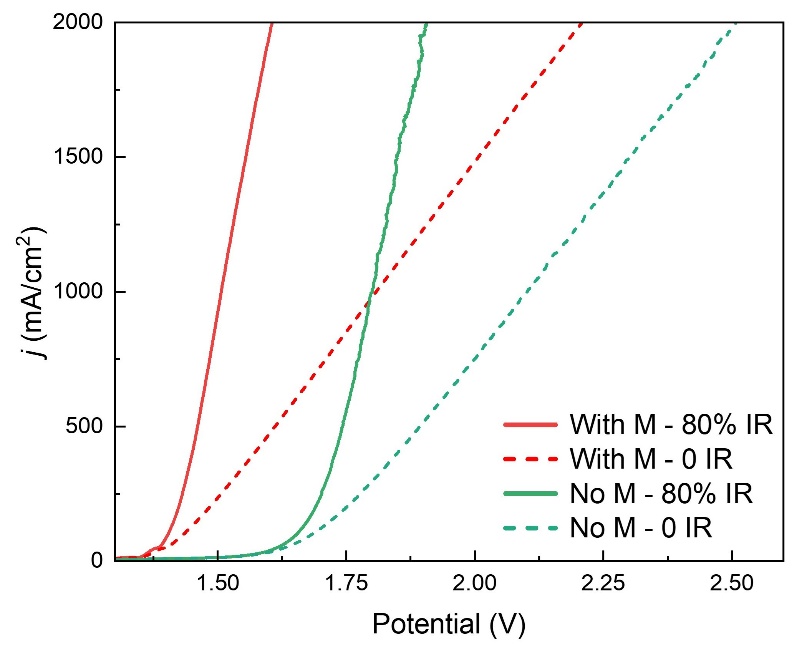


**Fig. S12 Both the IR-compensated (80% IR correction) and uncompensated raw data for the overall water-splitting performance of NiFeP MGWs.**


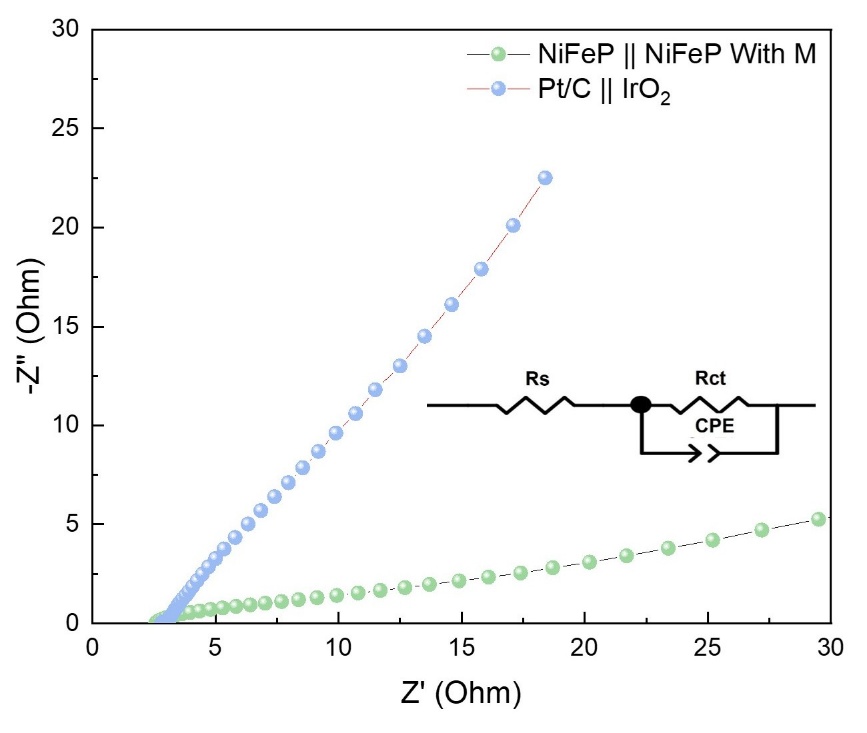


**Fig. S13 EIS result of NiFeP || NiFeP MGWs during overall water-splitting under magnetic field.**


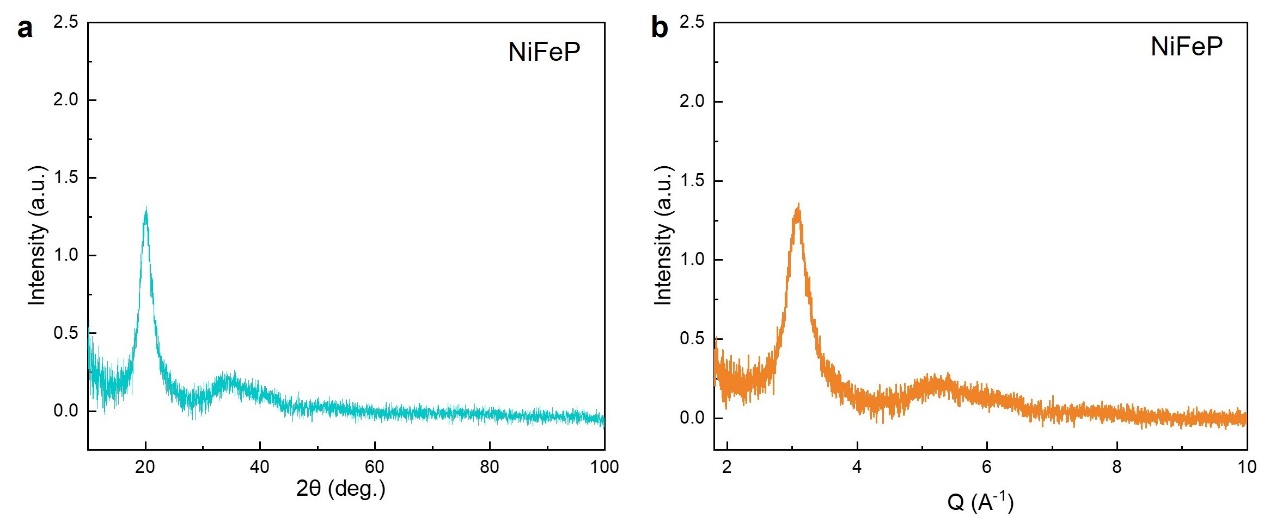


**Fig. S14 Fine XRD analysis results of** **NiFeP MGWs.**


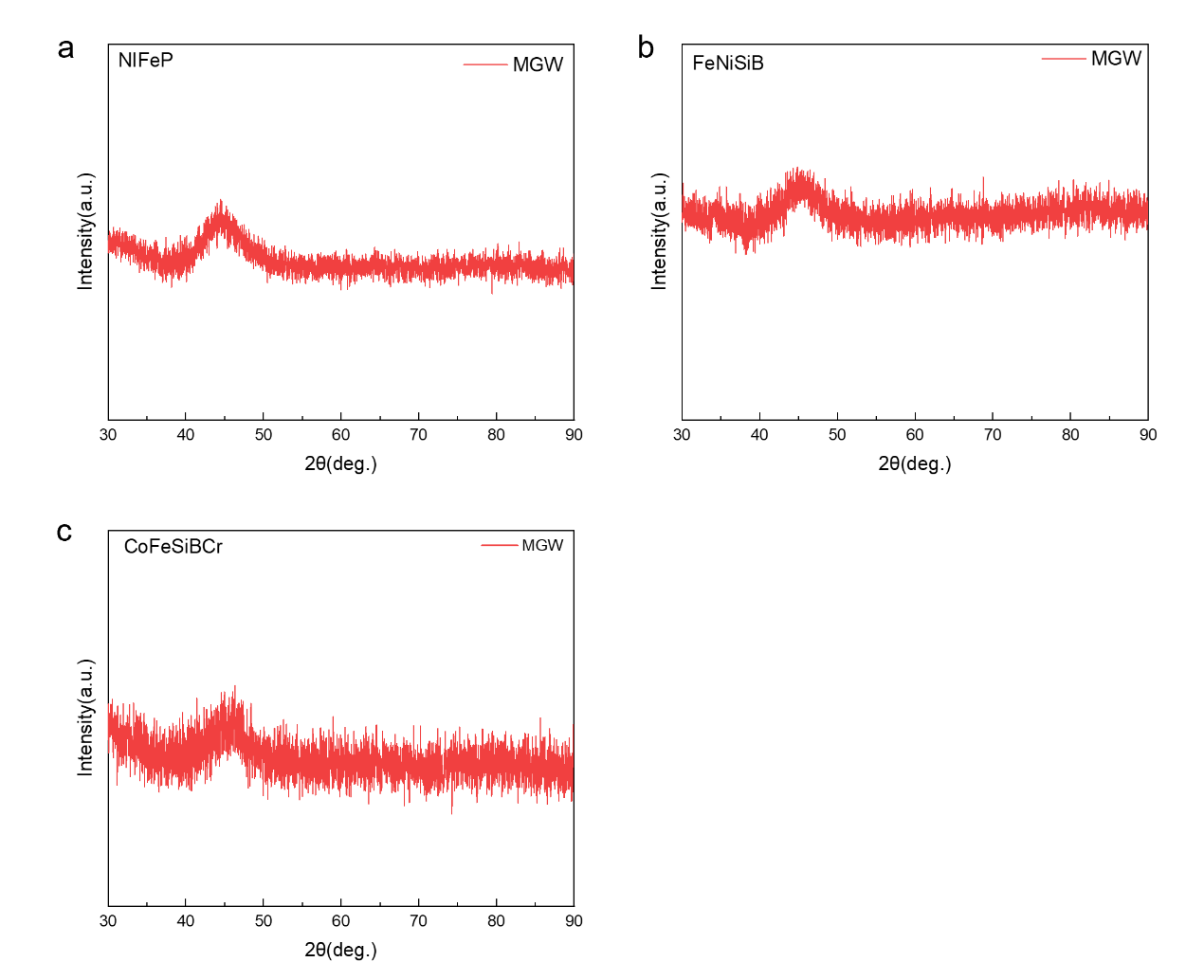


**Fig. S15 XRD results of NiFeP, FeNiSiB and CoFeSiBCr MGWs.**


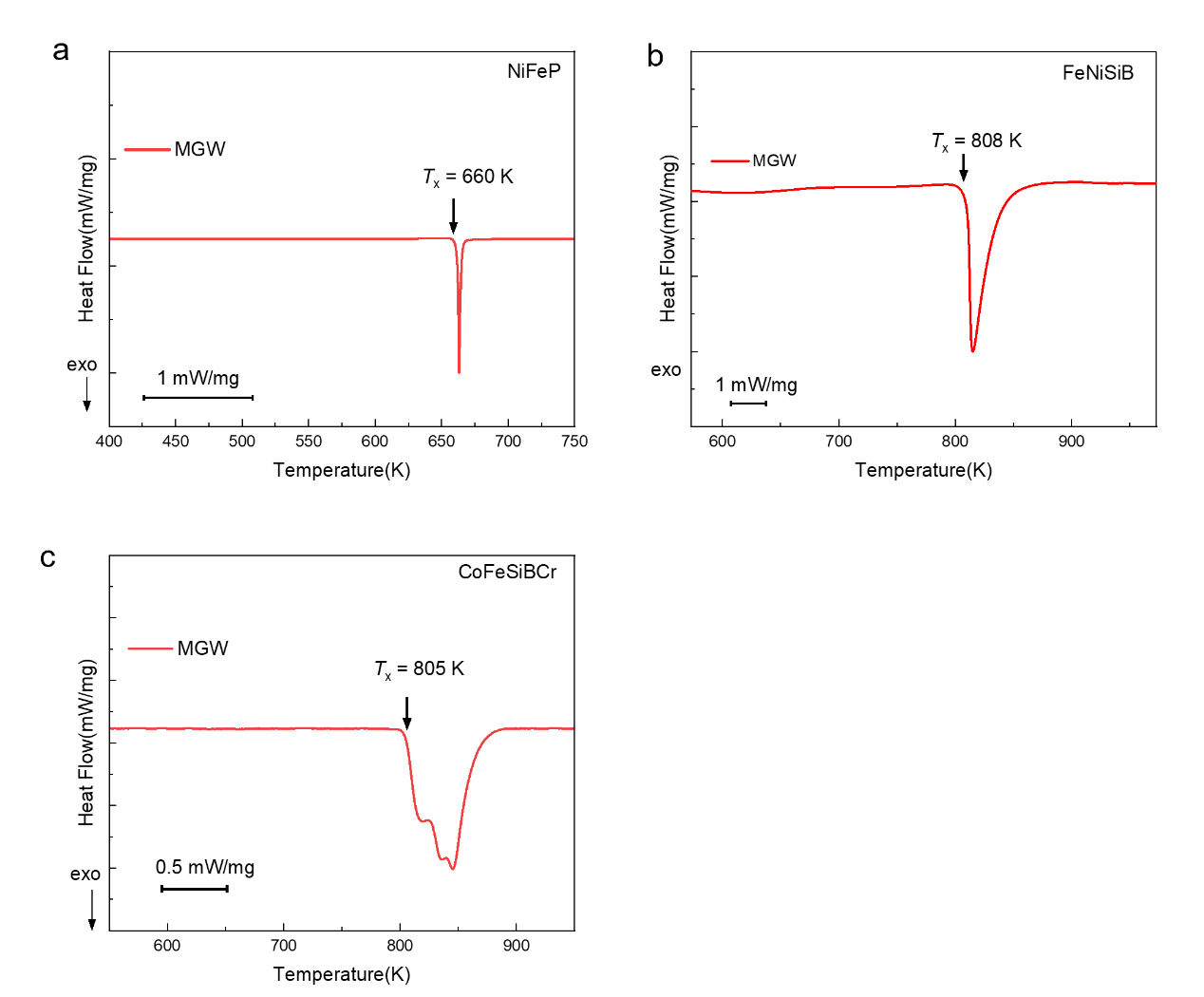


**Fig. S16 DSC results of NiFeP, FeNiSiB and CoFeSiBCr MGWs.**


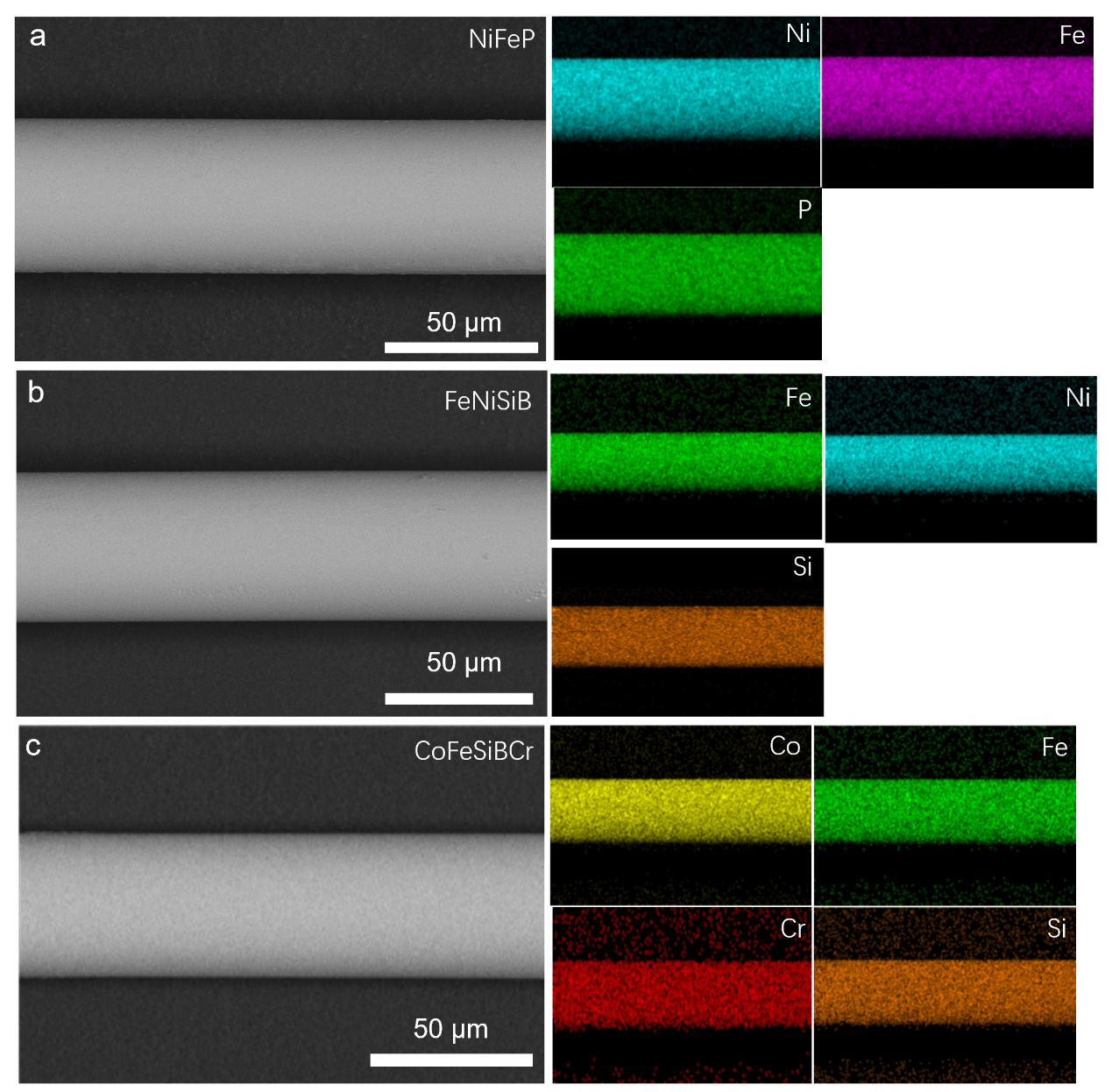


**Fig. S17 SEM and corresponding EDS mapping results of NiFeP, FeNiSiB and CoFeSiBCr MGWs.**


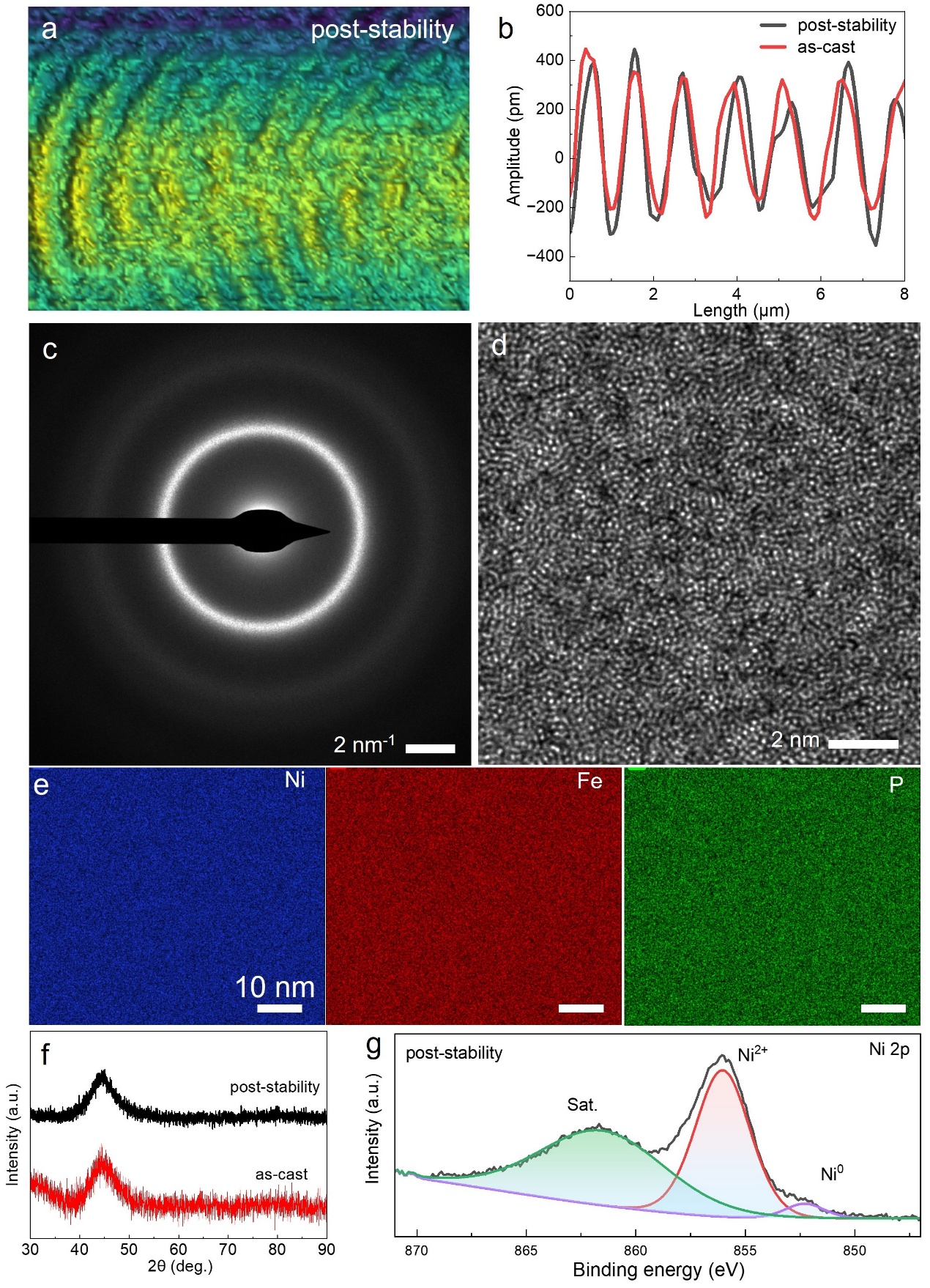


**Fig. S18 Structure characterizations of post-stability of NiFeP MGWs.**


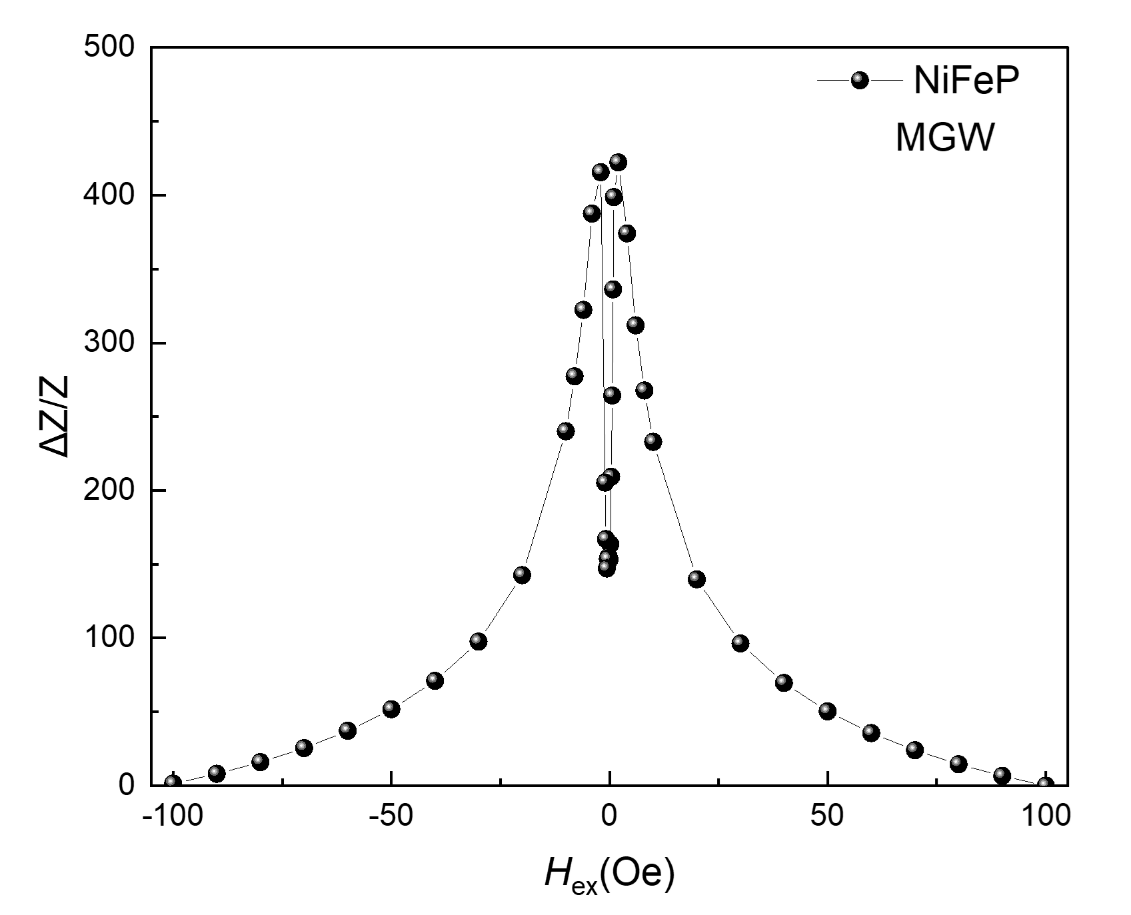


**Fig. S19 GMI result of NiFeP MGWs.**


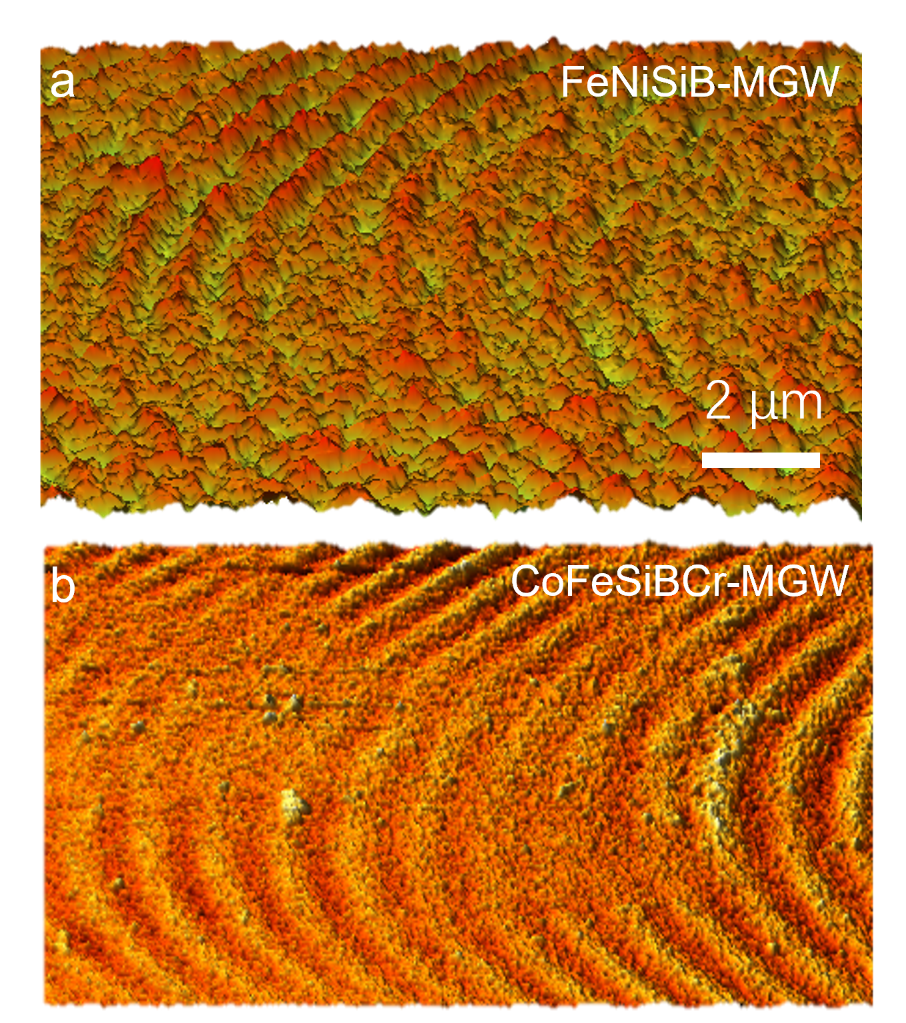


**Fig. S20 3D-magnetic domain structure results of FeNiSiB and CoFeSiBCr MGWs.**


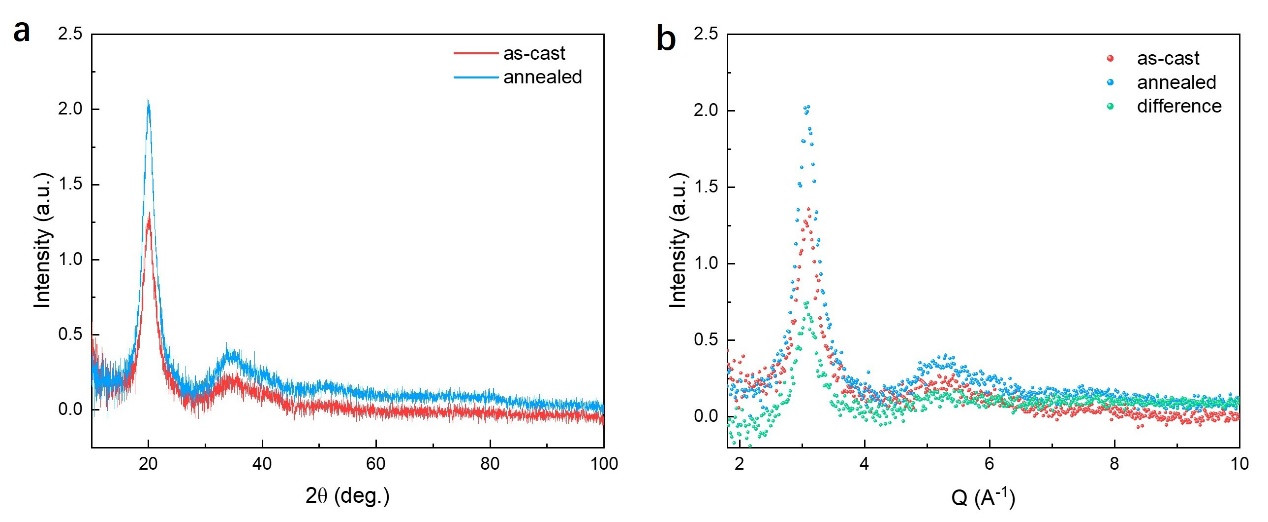


**Fig. S21 Fine-XRD results of as-cast and annealed NiFeP MGWs.**


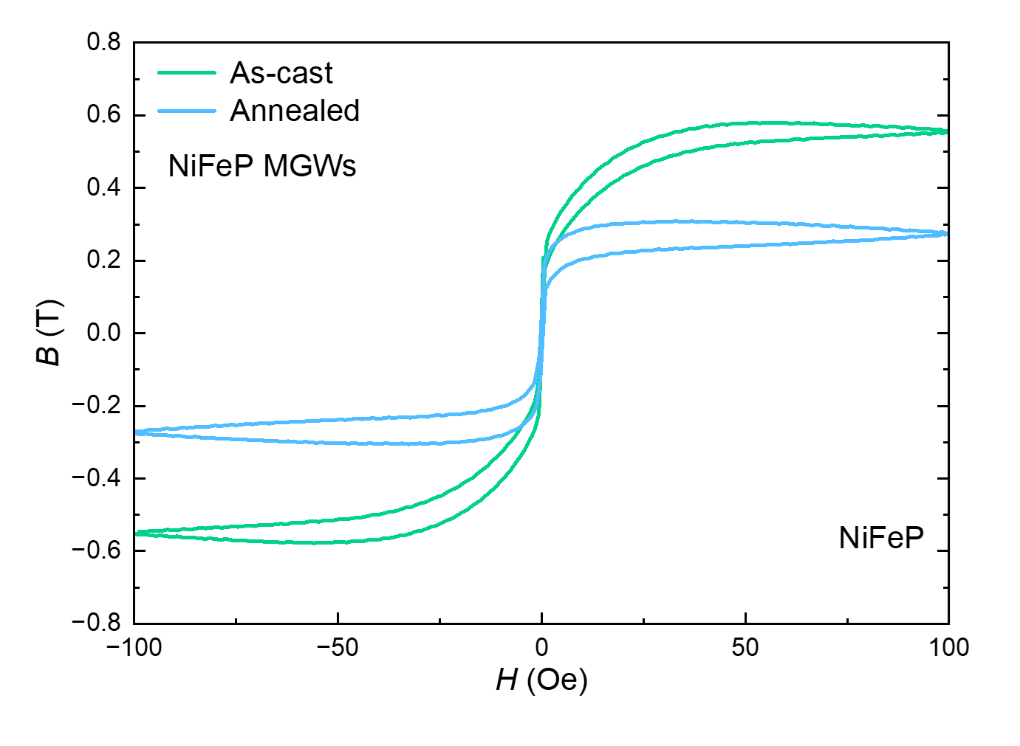


**Fig. S22 B-H results of as-cast and annealed NiFeP MGWs.**


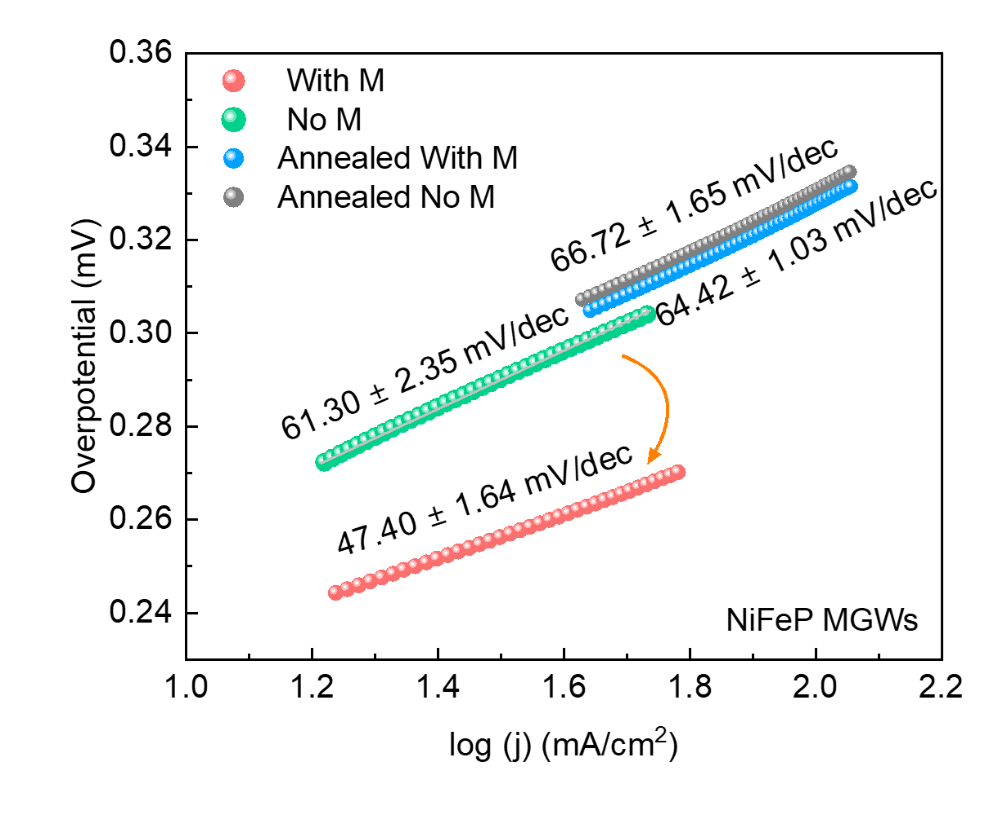


**Fig. S23 Tafel slopes of as-cast and annealed NiFeP MGWs with and without magnetic field.**

**Table S1 Fitted results for EIS spectra of NiFeP MGWs with and without magnetic fields.**

| Samples | *R_s_* (Ω) | *CPE* | *n* | *R_ct_* |
| --- | --- | --- | --- | --- |
| No M | 2.746 | 7.7074×10^-4^ | 0.831 | 205.9 |
| With M | 2.699 | 7.7019×10^-4^ | 0.817 | 172.6 |
| NiFeP \|\| NiFeP | 2.936 | 2.1771×10^-4^ | 0.686 | 25.5 |
| Pt/C \|\| IrO_2_ | 2.884 | 3.5936×10^-3^ | 0.691 | 53.9 |
